# Efficacy of pre-emptive kidney transplantation for adults with end-stage kidney disease: a systematic review and meta-analysis

**DOI:** 10.1080/0886022X.2023.2169618

**Published:** 2023-01-27

**Authors:** Tatsuhiko Azegami, Noriyuki Kounoue, Tadashi Sofue, Masahiko Yazawa, Makoto Tsujita, Kosuke Masutani, Yuki Kataoka, Hideyo Oguchi

**Affiliations:** a Keio University Health Center, Yokohama, Japan; b Department of Internal Medicine, Keio University School of Medicine, Tokyo, Japan; c Department of Nephrology, Toho University Faculty of Medicine, Tokyo, Japan; d Department of Cardiovascular and Cerebrovascular Medicine, Kagawa University, Takamatsu, Japan; e Department of Nephrology and Hypertension, St Marianna University School of Medicine, Kawasaki, Japan; f Department of Nephrology, Masuko Memorial Hospital, Nagoya, Japan; g Department of Internal Medicine, Faculty of Medicine, Division of Nephrology and Rheumatology, Fukuoka University, Fukuoka, Japan; h Department of Internal Medicine, Kyoto Min-Iren Asukai Hospital, Kyoto, Japan; i Scientific Research Works Peer Support Group (SRWS-PSG), Osaka, Japan; j Department of Community Medicine, Section of Clinical Epidemiology, Kyoto University Graduate School of Medicine, Kyoto, Japan; k Department of Healthcare Epidemiology, Kyoto University Graduate School of Medicine/School of Public Health, Kyoto, Japan

**Keywords:** Transplantation, meta-analysis, systematic review, pre-emptive kidney transplantation, mortality, graft failure

## Abstract

**Background:**

Pre-emptive kidney transplantation (PEKT), i.e., transplantation performed before initiation of maintenance dialysis, is considered an ideal renal replacement therapy because there is no exposure to long-term dialysis therapy. Therefore, we summarized advantages/disadvantages of PEKT to assist in deciding whether kidney transplantation should be performed pre-emptively.

**Methods:**

This study was registered with PROSPERO, CRD42021269163. Observational studies comparing clinical outcomes between PEKT and non-PEKT were included; those involving only pediatric recipients or simultaneous multi-organ transplantations were excluded. The PubMed/MEDLINE, Cochrane Library, and Ichushi-Web databases were searched on 1 August 2021. Studies were pooled using the generic inverse-variance method with random effects model, and risk of bias was assessed using ROBINS-I.

**Results:**

Seventy-six studies were included in the systematic review (sample size, 23–121,853; enrollment year, 1968–2019). PEKT patients had lower all-cause mortality (adjusted HR: 0.78 [95% CI 0.66–0.92]), and lower death-censored graft failure (0.81 [0.67–0.98]). Unadjusted RRs for the following outcomes were comparable between the two patient groups: cardiovascular disease, 0.90 (0.58–1.40); biopsy-proven acute rejection, 0.75 (0.55–1.03); cytomegalovirus infection, 1.04 (0.85–1.29); and urinary tract infection, 0.89 (0.61–1.29). Mean differences in post-transplant QOL score were comparable in both groups. The certainty of evidence for mortality and graft failure was moderate and that for other outcomes was very low following the GRADE classification.

**Conclusions:**

The present meta-analysis shows the potential benefits of PEKT, especially regarding patient and graft survival, and therefore PEKT is recommended for adults with end-stage kidney disease.

## Introduction

Chronic kidney disease (CKD) is usually an irreversible and progressive disease that can lead to end-stage kidney disease (ESKD), which is a significant risk factor for death and cardiovascular disease (CVD) [1]. Most patients with ESKD require renal replacement therapy via hemodialysis, peritoneal dialysis, or kidney transplantation. Although the mortality rate has declined among dialysis patients in recent decades [2,3], it remains high compared with that of the general population [4]. Kidney transplantation is preferable to dialysis because it is associated with superior survival, cardiovascular outcome, and quality of life (QOL) and has a lower cost than dialysis [5–8].

Previous studies have indicated that longer waiting time on pre-transplant dialysis is a strong risk factor for death [9,10]. Therefore, pre-emptive kidney transplantation (PEKT), i.e., transplantation performed before initiation of maintenance dialysis, is considered the ideal and optimal treatment for most patients with ESKD because of no exposure to long-term dialysis therapy. Although PEKT is generally recommended when the glomerular filtration rate falls below 15 mL/min, the optimal timing for PEKT is still unclear [11]. The Descartes Working Group and the European Renal Best Practice (ERBP), which are both official bodies of the ERA-EDTA (European Renal Association-European Dialysis and Transplant Association), recommend that PEKT is planned in order to avoid dialysis, based on the results of their systematic literature review in 2016 on PEKT limited to living donor transplantation [11]. However, only 2.5% of patients with ESKD in the United States and 4% of patients with ESKD in European countries actually receive a pre-emptive transplant [12,13].

As living donor transplantations account for only 31.7% of all kidney transplantations [14], both living and deceased donor transplantations need to be included in order to systematically evaluate the usefulness of PEKT. Furthermore, a large number of papers on PEKT have been published since the publication of the ERA-EDTA recommendation in 2016, and there is a strong need to collect together the findings of these recent studies and update the evidence concerning the clinical effects of PEKT. In addition, whether kidney transplantation should be performed pre-emptively is an important clinical question; however, no meta-analysis has been conducted to evaluate the clinical efficacy of PEKT.

We therefore conducted a systematic review of the literature on kidney transplantation from living and deceased donors and meta-analyses to summarize the advantages and disadvantages of PEKT over non-PEKT. We hope that understanding the prognostic characteristics of PEKT will help clinical physicians to decide whether kidney transplantation should be performed pre-emptively in their medical practice.

## Materials and methods

This systematic review and meta-analysis was performed according to the Preferred Reporting Items for Systematic reviews and Meta-Analyses (PRISMA) statement and the Meta-analysis of Observational Studies in Epidemiology (MOOSE) reporting guideline [15,16]. The protocol for the review was registered and published on International Prospective Register of Systematic Reviews (PROSPERO) (ID: CRD42021269163).

### Eligibility criteria

Studies were eligible for inclusion if they had examined the important clinical outcomes described in the ‘Data collection process’ section in both PEKT and non-PEKT patients. PEKT was defined as kidney transplantation performed before initiation of maintenance dialysis, while non-PEKT was defined as kidney transplantation after initiation of maintenance dialysis. All adults with ESKD eligible for kidney transplantation, regardless of the type of donor (living or deceased) were included in the present study. Studies involving only pediatric recipients (age <18 years) and those involving only simultaneous multi-organ transplantations were excluded. Non-English and non-Japanese articles were included for languages where an appropriate translator was available. Although we would like to aim to include randomized controlled trials (RCTs), it is almost impossible for ethical reasons to conduct RCTs comparing the outcome after PEKT vs. non PEKT. Therefore, we included only observational studies.

### Information sources and search strategy

A systematic electronic search was performed in PubMed/MEDLINE, The Cochrane Library, and Ichushi-Web, which is an online database of articles published in Japanese-language medical journals, on 1 August 2021. In addition, we hand-searched the reference list of a relevant systematic review [11] for additional studies and confirmed that all studies were included in the results list of the initial database search. The detailed search strategies are included in Supplemental Table 1. Two authors (T.A. and N.K.) independently searched the database following the advice of experienced searchers, and any studies considered potentially relevant by at least one reviewer was recovered for further review.

**Table 1.** Overview of studies included in the systematic review.

Author	Year	Country	Population	Arms	Sample size	Age, years	Sex, percentage of males (%)	Donor source, percentage of LD (%)	Dialysis duration
Auneau-Enjalbert [1]	2021	France	>18 years old	PEKT	178	57 ± 14 (mean ± SD)	65	39	–
				Non-PEKT	196	55 ± 15 (mean ± SD)	66	30	13.5 ± 9.1 months (mean ± SD)
Aytekin [2]	2020	Turkey		PEKT	218	36.30 ± 16.27 (mean ± SD)	64.7	N.A.	–
				Non-PEKT	448	35.20 ± 15.54 (mean ± SD)	66.3	N.A.	N.A.
Mitsui [3]	2020	Japan	LD transplantation	PEKT	12	43.5, 19–65 (median, range)	75	100	–
				Non-PEKT	20	43.5, 17–68 (median, range)	65	100	1.37, 0.17–6.9 years (median, range)
Franco [4]	2020	Spain	DD transplantation	PEKT	66	51.2, 48.0–54.4 (mean, 95% CI)	62.1	0	–
				Non-PEKT (matched)	66	51.8, 49.0–54.7 (mean, 95% CI)	62.1	0	N.A.
Irish [5]	2019	Australia, New Zealand	LD transplantation	PEKT	699	43.0, 31.0–54.0 (median, IQR)	65.3	100	–
			Adults	Non-PEKT (matched)	699	43.0, 31.0–53.0 (median, IQR)	64.6	100	≤6 months
Kim [6]	2019	Korea	LD transplantation	PEKT (dialysis <19 months)	493	46.0 ± 12.2 (mean ± SD)	65.5	100	3.0, 0–18 months (median, IQR)
				Non-PEKT (matched)	493	45.2 ± 11.9 (mean ± SD)	63.7	100	48.0, 19–288 months (median, IQR)
Foucher [7]	2019	France	DD transplantation	PEKT	554	52.8 ± 15.0 (mean ± SD)	58.7	0	–
			Adults	Non-PEKT	584	51.9 ± 12.7 (mean ± SD)	58.7	0	N.A.
Prezelin-Reydit [8]	2019	France	Adults	PEKT	3112	48.8 ± 13.8 (mean ± SD)	59.0	22.2	–
				Non-PEKT	19,176	50.8 ± 13.3 (mean ± SD)	62.4	7.0	2.3, 1.3–4.1 years (median, IQR)
Mochizuki [9]	2019	Japan	LD transplantation	PEKT	18	42.5, 12–65 (median, range)	66.7	100	–
				Non-PEKT	52	45.0, 9–72 (median, range)	61.5	100	28 months
Matsumura [10]	2018	Japan	LD transplantation	PEKT	50	49.5 ± 14.2 (mean ± SD)	66	100	–
			>20 years old	Non-PEKT	49	47.0 ± 14.3 (mean ± SD)	73	100	58.4 ± 63.6 months (mean ± SD)
Aufhauser [11]	2018	United States	DD transplantation	PEKT	10,360	55, 46–63 (median, IQR)	54	0	–
			≥18 years old	Non-PEKT, <5 years dialysis	72,723	54, 43–62 (median, IQR)	62	0	<5 years
				Non-PEKT, 5–9 years dialysis	22,894	52, 42–60 (median, IQR)	60	0	5–9 years
				Non-PEKT, 10–14 years dialysis	2473	49, 40–58 (median, IQR)	44	0	10–14 years
				Non-PEKT, 15–19 years dialysis	451	49, 40–57 (median, IQR)	41	0	15–19 years
				Non-PEKT, ≥20 years dialysis	178	51, 38–64 (median, IQR)	45	0	≥20 years
Gill [12]	2018	United States	LD transplantation	PEKT	26,217	50, 39–59 (median, IQR)	59.8	100	–
			≥18 years old	Non-PEKT	51,390	48, 36–58 (median, IQR)	62.9	100	14, 8–27 months (median, IQR)
Mursawa [13]	2018	Japan		PEKT	19	38, 34–42 (median, IQR)	57.9	100	–
				Non-PEKT	81	47, 36–56 (median, IQR)	63.0	64	22, 9–85 months (median, IQR)
Girerd [14]	2018	France	Second transplantation	PEKT	93	45.7 ± 13.8 (mean ± SD)	58.1	29.0	–
				Non-PEKT	1221	47.1 ± 13.4 (mean ± SD)	62.0	7.0	39.2, 19.5–74.7 months (median, IQR)
Haller [15]	2017	Austria		PEKT	461	39 ± 17 (mean ± SD)	65	56	–
				Non-PEKT, first tertile	2124	46 ± 16 (mean ± SD)	65	19	<1.5 years
				Non-PEKT, second tertile	2119	52 ± 15 (mean ± SD)	64	4	1.5–3.1 years
				Non-PEKT, third tertile	2186	51 ± 13 (mean ± SD)	63	1	>3.1 years
Okumi [16]	2017	Japan	LD transplantation	PEKT	93	43.4 ± 14.0 (mean ± SD)	62.4	100	–
			≥18 years old	Non-PEKT (matched)	93	43.6 ± 12.1 (mean ± SD)	64.5	100	24, 12–55 months (median, IQR)
Nakagawa [17]	2017	Japan		PEKT	2234	40.4 ± 16.6	N.A.	N.A.	–
				Non-PEKT	10,642	42.6 ± 15.5	N.A.	N.A.	N.A.
Gadelkareem [18]	2017	Egypt	LD transplantation	PEKT	30	44.1 ± 12.1 (mean ± SD)	66.7	100	–
				Non-PEKT	15	34.3 ± 14.6 (mean ± SD)	60.0	100	≤6 months
Girerd [19]	2016	France	Second transplantation	PEKT	22	47.3 ± 11.5 (mean ± SD)	N.A.	9.1	–
			>18 years old	Non-PEKT	224	44.6 ± 12.9 (mean ± SD)	N.A.	7.4	47.2 months (mean)
Bzoma [20]	2016	Poland	Receiving a graft from same donor	PEKT	23	50, 24–69 (mean, range)	52.2	0	–
				Non-PEKT	23	53, 31–76 (mean, range)	60.9	0	39.5, 2.5–121 months (mean, range)
Goto [21]	2016	Japan	LD transplantation	PEKT	239	43.1 ± 14.2 (mean ± SD)	62.3	100	–
			>18 years old	Non-PEKT	547	45.7 ± 13.8 (mean ± SD)	63.1	100	N.A.
Jay [22]	2016	United States	LD transplantation	PEKT	14,503	47 ± 15 (mean ± SD)	59	100	–
				Non-PEKT, <1 year dialysis	7590	43 ± 16 (mean ± SD)	63	100	<1 year
				Non-PEKT, ≥1 year dialysis	17,503	46 ± 15 (mean ± SD)	61	100	≥1 year
Noda [23]	2016	Japan	LD transplantation	PEKT	7	58, 36–75 (median, range)	71.4	100	–
				Non-PEKT	16	52, 13–69 (median, range)	43.8	100	39.5, 2–110 months (median, range)
Florit [24]	2015	Spain	Second transplantation	PEKT	18	45 (mean)	N.A.	88.9	–
				Non-PEKT	83	55 (mean)	N.A.	19.3	N.A.
Morales [25]	2015	Spain	DD transplantation	PEKT	26	74.3 ± 2.9 (mean ± SD)	57.7	0	–
			>65 years old	Non-PEKT	26	73.4 ± 4.1 (mean ± SD)	50.0	0	15 ± 14 months (mean ± SD)
Unsal [26]	2015	Turkey	Adults	PEKT	90	37.9 ± 10.4 (mean ± SD)	57.8	10.0	–
				Non-PEKT	244	41.5 ± 12.8 (mean ± SD)	61.1	22.1	27 ± 38 months (mean ± SD)
Nakamura [27]	2015	Japan	LD transplantation	PEKT (dialysis 0 months)	64	38 ± 17.0 (mean ± SD)	70.3	100	–
				Non-PEKT (dialysis >120 months)	18	51 ± 10.7 (mean ± SD)	50.0	100	>120 months
Oishi [28]	2015	Japan		PEKT	25	43.6 (range, 10–72)	64.0	100	–
				Non-PEKT	61	49.3 (range, 16–76)	59.0	86.9	66 months
Dębska-Ślizień [29]	2014	Poland	Receiving a graft from same donor	PEKT	51	42.0 ± 14.0 (mean ± SD)	43.1	0	–
				Non-PEKT	51	47.5 ± 13.6 (mean ± SD)	68.6	0	3.2 ± 3.0 years (mean ± SD)
Kohei [30]	2014	Japan	LD transplantation	PEKT	23	35.5 ± 13.3 (mean ± SD)	78.2	100	–
			≥18 years old	Non-PEKT	403	40.2 ± 13.5 (mean ± SD)	62.5	100	≤24 months
				Non-PEKT	346	43.0 ± 13.2 (mean ± SD)	61.3	100	25–60 months
				Non-PEKT	203	44.7 ± 13.4 (mean ± SD)	60.1	100	61–120 months
				Non-PEKT	110	44.9 ± 11.8 (mean ± SD)	59.1	100	121–240 months
				Non-PEKT	13	51.3 ± 8.9 (mean ± SD)	53.8	100	≥241 months
Ryosaka [31]	2014	Japan		PEKT	65	40.6 ± 12.5 (mean ± SD)	61.5	98.5	–
				Non-PEKT	708	46.2 ± 13.6 (mean ± SD)	62.3	92.1	N.A.
Sayin [32]	2013	Turkey		PEKT	37	34.02 ± 10.61 (mean ± SD)	89.2	97.3	–
				Non-PEKT	63	31.44 ± 10.41 (mean ± SD)	74.6	73.0	24 ± 18 months (mean ± SD)
Bozkurt [33]	2013	Turkey		PEKT	153	34.5 (mean)	74.5	97.4	–
				Non-PEKT	706	37.7 (mean)	62.4	73.0	N.A.
Johnston [34]	2013	United States	Second transplantation	PEKT	3509	N.A.	54.7	57.6	–
			≥18 years old	Non-PEKT	14,075	N.A.	58.4	26.9	N.A.
Grams [35]	2013	United States	DD transplantation	PEKT	10,992	52.7 ± 12.5 (mean ± SD)	N.A.	0	–
				Non-PEKT, ≤1 year dialysis	14,428	50.6 ± 13.2 (mean ± SD)	N.A.	0	≤1 year
				Non-PEKT, >1 year dialysis	96,433	50.9 ± 13.0 (mean ± SD)	N.A.	0	>1 year
Hayashida [36]	2013	Japan	LD transplantation	PEKT	29	49, 18–69 (median, range)	80	100	–
				Non-PEKT (dialysis ≥5 years)	15	51, 37–63 (median, range)	72	100	94, 61–285 months (median, range)
Luo [37]	2012	China	DD transplantation	PEKT	32	41.36 ± 8.87 (mean ± SD)	59.4	0	–
			Adults	Non-PEKT	132	42.05 ± 10.69 (mean ± SD)	65.2	0	14.65 ± 7.53 months (mean ± SD)
Keith [38]	2012	United States	DD transplantation	PEKT	N.A.	N.A.	N.A.	0	–
				Non-PEKT	N.A.	N.A.	N.A.	0	29.0 ± 30.5, Caucasian; 45.6 ± 35.1 months, others (mean ± SD)
Naveed [39]	2011	United States	ESKD due to SLE	PEKT	730	40.0 ± 11.6 (mean ± SD)	17.5	N.A.	–
				Non-PEKT	7271	36.9 ± 11.7 (mean ± SD)	18.6	N.A.	N.A.
Rigo [40]	2011	Argentina	≥18 years old	PEKT	28	29 (mean)	48	N.A.	–
				Non-PEKT, LD	27	30 (mean)	57	100	N.A.
				Non-PEKT, DD	25	35 (mean)	68	0	N.A.
Kessler [41]	2011	France	DD transplantation	PEKT	118	44.3 ± 12.9 (mean ± SD)	51.7	0	–
			≥18 years old	Non-PEKT	1467	48.4 ± 13.4 (mean ± SD)	62.8	0	3.40 ± 3.21 years (mean ± SD)
Son [42]	2010	Korea	LD transplantation	PEKT	30	50.6 ± 10.5 (mean ± SD)	66.7	100	–
			Diabetic ESKD patient	Non-PEKT, HD	22	49.6 ± 10.8 (mean ± SD)	77.3	100	18.3 ± 14.0 months (mean ± SD)
				Non-PEKT, PD	18	48.5 ± 12.5 (mean ± SD)	56.6	100	20.4 ± 15.4 months (mean ± SD)
Jung [43]	2010	Korea	LD transplantation	PEKT	62	41.6 ± 9.9 (mean ± SD)	54.8	100	–
			≥15 years old	Non-PEKT	390	40.1 ± 10.7 (mean ± SD)	62.1	100	25.6 ± 34.6 months (mean ± SD)
Witczak [44]	2009	Norway		PEKT	809	44.7 ± 17.2 (mean ± SD)	62	64	–
				Non-PEKT	2591	52.0 ± 16.4 (mean ± SD)	67	35	14.4 ± 12.8 months (mean ± SD)
Salvadori [45]	2009	Italy	DD transplantation	PEKT	43, all (17, transplanted)	48 ± 12.5, all (mean ± SD)	73, all	0	–
				Non-PEKT	120, all (41, transplanted)	47 ± 12.9, all (mean ± SD)	71.6, all	0	21.3 ± 17.8 months, all (mean ± SD)
Yoo [46]	2009	Korea	LD transplantation	PEKT	81	37.5 ± 9.5 (mean ± SD)	51.9	100	–
			≥15 years old	Non-PEKT, HD	343	39.0 ± 10.8 (mean ± SD)	65.9	100	29.3 ± 36.9 months (mean ± SD)
				Non-PEKT, PD	75	39.1 ± 10.9 (mean ± SD)	69.3	100	33.1 ± 34.2 months (mean ± SD)
Milton [47]	2008	Australia	LD transplantation	PEKT	578	35.0, 33.7–36.4 (mean, 95% CI)	N.A.	100	–
				Non-PEKT	2025	37.7, 37.0–38.4 (mean, 95% CI)	N.A.	100	N.A.
Ishikawa [48]	2008	Japan	LD transplantation	PEKT	5	32.0 ± 13.2 (mean ± SD)	40.0	100	–
				Non-PEKT	39	40.8 ± 13.3 (mean ± SD)	64.1	100	42.4 ± 41.2 months (mean ± SD)
Joo [49]	2007	Korea	LD transplantation	PEKT	63	40.8 ± 11.5 (mean ± SD)	57.1	100	–
			≥18 years old	Non-PEKT, HD	359	36.6 ± 11.1 (mean ± SD)	68.0	100	13.9 ± 23.0 months (mean ± SD)
				Non-PEKT, PD	72	39.1 ± 12.2 (mean ± SD)	61.1	100	17.5 ± 17.6 months (mean ± SD)
Pour-Reza-Gholi [50]	2007	Iran	LD transplantation	PEKT	300	29.4 ± 17.2 (mean ± SD)	56.6	100	–
				Non-PEKT	300	34.2 ± 15.5 (mean ± SD)	57.3	100	15.70 ± 14.56 months (mean ± SD)
Pérez-Flores [51]	2007	Spain	DD transplantation	PEKT	33	48 ± 14 (mean ± SD)	N.A.	0	–
				Non-PEKT	387	49 ± 13 (mean ± SD)	N.A.	0	N.A.
Innocenti [52]	2007	United States	LD transplantation	PEKT	191	48.9 ± 15.9 (mean ± SD)	50.8	100	–
				Non-PEKT	247	46.5 ± 15.5 (mean ± SD)	60.3	100	21 ± 36 months (mean ± SD)
Kennedy [53]	2006	Australia	<30 years old	PEKT	N.A.	N.A.	N.A.	N.A.	–
				Non-PEKT	N.A.	N.A.	N.A.	N.A.	N.A.
Dębska-Ślizień [54]	2006	Poland		PEKT	15	40.0 ± 14.8 (mean ± SD)	46.7	13.3	–
				Non-PEKT	115	45.6 ± 13.2 (mean ± SD)	67.0	0	41 ± 38 months (mean ± SD)
Goldfarb-Rumyantzev [55]	2006	United States	Second transplantation	PEKT	1609	38.5 ± 13.0 (mean ± SD)	57.4	30.0	–
				Non-PEKT	10,105	38.5 ± 12.7 (mean ± SD)	59.8	17.6	25.0, 10.9–48.6 months (median, IQR)
Becker [56]	2006	United States	Diabetic ESKD patient	PEKT	2476	N.A.	N.A.	N.A.	–
			≥18 years old	Non-PEKT	20,762	N.A.	N.A.	N.A.	N.A.
Abou Ayache [57]	2005	France	Adults	PEKT	44	48.5 ± 12 (mean ± SD)	61.4	15.9	–
				Non-PEKT	419	44.6 ± 14 (mean ± SD)	60.4	2.1	N.A.
Goldfarb-Rumyantzev [58]	2005	United States		PEKT	N.A.	N.A.	N.A.	N.A.	–
				Non-PEKT	N.A.	N.A.	N.A.	N.A.	2.2 ± 2.2 years (mean ± SD)
Gill [59]	2004	Canada	18–70 years old	PEKT, LD	2999	38 ± 11 (mean ± SD)	55	100	–
				PEKT, DD	2967	44 ± 12 (mean ± SD)	58	0	–
				Non-PEKT, LD	8291	39 ± 12 (mean ± SD)	59	100	N.A.
				Non-PEKT, DD	26,706	45 ± 12 (mean ± SD)	61	0	N.A.
el-Agroudy [60]	2004	Egypt	LD transplantation	PEKT	82	27.9 ± 10.1 (mean ± SD)	63	100	–
				Non-PEKT	1197	29.9 ± 10.3 (mean ± SD)	76	100	N.A.
Simforoosh [61]	2003	Iran	LD transplantation	PEKT	127	26.63 ± 14.78 (mean ± SD)	64.6	100	–
				Non-PEKT	186	29.66 ± 14.02 (mean ± SD)	66.7	100	19.46 ± 17.55 months (mean ± SD)
Mange [62]	2003	United States	LD transplantation	PEKT	1819	40.0 ± 12 (mean ± SD)	53.4	100	–
			≥18 years old	Non-PEKT	6662	41.0 ± 13 (mean ± SD)	58.8	100	329 days (median)
Nishikawa [63]	2002	United States		PEKT, LD	8480	N.A.	N.A.	100	–
				PEKT, DD	4101	N.A.	N.A.	0	–
				Non-PEKT, LD	25,061	N.A.	N.A.	100	N.A.
				Non-PEKT, DD	52,518	N.A.	N.A.	0	N.A.
Meier-Kriesche [64]	2002	United States	Recipients of paired kidneys	PEKT (dialysis <6 months)	2405	44.3 ± 12.8 (mean ± SD)	59.8	0	1.1 ± 1.9 months (mean ± SD)
				Non-PEKT (dialysis >24 months)	2405	47.3 ± 12.5 (mean ± SD)	58.6	0	51.2 ± 34.6 months (mean ± SD)
Kasiske [65]	2002	Canada		PEKT, LD	3141	N.A.	N.A.	N.A.	–
				PEKT, DD	1977	N.A.	N.A.	N.A.	
				Non-PEKT, LD	9937	N.A.	N.A.	N.A.	
				Non-PEKT, DD	23,781	N.A.	N.A.	N.A.	
Mange [66]	2001	United States	LD transplantation	PEKT	1819	40 ± 12 (mean ± SD)	53.4	100	–
				Non-PEKT	6662	41 ± 13 (mean ± SD)	58.8	100	329 ± 638 days (mean ± SD)
Meier-Kriesche [67]	2000	United States		PEKT	N.A.	N.A.	N.A.	N.A.	–
				Non-PEKT	N.A.	N.A.	N.A.	N.A.	N.A.
Papalois [68]	2000	United States		PEKT, LD	313	32.6 (mean)	N.A.	100	–
				PEKT, DD	72	39.1 (mean)	N.A.	0	–
				Non-PEKT, LD	761	34.7 (mean)	N.A.	100	N.A.
				Non-PEKT, DD	703	44.5 (mean)	N.A.	0	N.A.
John [69]	1998	India	LD transplantation	PEKT	43	32.5 ± 12.2 (mean ± SD)	74.4	100	–
				Non-PEKT (matched)	86	32.3 ± 11.5 (mean ± SD)	74.4	100	2.8 ± 1.4 months (mean ± SD)
Asderakis [70]	1998	United Kingdom		PEKT	161	49.7 (mean)	62	14.2	–
				Non-PEKT	1302	50.1 (mean)	64.3	7.3	N.A.
Roake [71]	1996	United Kingdom	DD transplantation	PEKT	116	44.2 ± 12.9 (mean ± SD)	55.2	0	–
				Non-PEKT (matched)	116	44.5 ± 12.4 (mean ± SD)	55.2	0	8, 1–298 months (median, range)
Berthoux [72]	1996	ERA-EDTA registry		PEKT	2248	N.A.	N.A.	31.6	–
				Non-PEKT, <1 year dialysis	12,180	N.A.	N.A.	21.0	<1 year
				Non-PEKT, 1–5 years dialysis	16,283	N.A.	N.A.	5.9	1–5 years
				Non-PEKT, >5 years dialysis	594	N.A.	N.A.	2.8	>5 years
Ekstrand [73]	1993	Finland	ESKD due to diabetic nephropathy	PEKT	24	39 ± 2 (mean ± SEM)	N.A.	12	–
				Non-PEKT	101	36 ± 1 (mean ± SEM)	N.A.	5	6.8 months (mean)
Cacciarelli [74]	1993	United States		PEKT, LD	22	38 ± 11 (mean ± SD)	36	100	–
				PEKT, DD	15	40 ± 16 (mean ± SD)	60	0	–
				Non-PEKT, LD (HD)	120	38 ± 12 (mean ± SD)	71	100	N.A.
				Non-PEKT, LD (PD)	36	29 ± 16 (mean ± SD)	47	100	N.A.
				Non-PEKT, DD (HD)	408	41 ± 13 (mean ± SD)	61	0	N.A.
				Non-PEKT, DD (PD)	61	36 ± 15 (mean ± SD)	57	0	N.A.
Katz [75]	1991	United States		PEKT	85	33.0 ± 1.3 (mean ± SEM)	55.3	55.3	–
				Non-PEKT (matched)	84	34.3 ± 1.3 (mean ± SEM)	57.1	48.8	N.A.
Migliori [76]	1987	United States		PEKT	132	N.A.	N.A.	72.7	–
				Non-PEKT, ≤30 days dialysis	206	N.A.	N.A.	87.4	≤30 days
				Non-PEKT, >30 days dialysis	1404	N.A.	N.A.	55.6	>30 days

CI: confidential interval; DD: deceased donor; ESKD: end-stage kidney disease; HD: hemodialysis; IQR: interquartile range; LD: living donor; N.A.: not available; PD: peritoneal dialysis; PEKT: preemptive kidney transplantation; SD: standard deviation; SEM: standard error of mean; SLE: systemic lupus erythematosus.

References (numbers in [ ]) are listed in Supplemental Item 5.

### Selection process

As a primary screening, titles and abstracts were independently screened by two authors (T.A. and N.K.). In the secondary screening that followed, the full text of each potentially relevant study was independently assessed by two authors (T.A. and N.K.) for inclusion in the present systematic review. Unpublished data and reports from conference abstracts were excluded from the systematic review. Disagreements between reviewers were resolved by a consensus-based discussion.

### Data collection process

Data from included studies were extracted by two authors (T.A. and N.K.). We contacted study authors via e-mail for additional information where necessary. The detailed list of relevant items collected during data extraction is available in Online Resource 1. The primary outcome was patient survival. Secondary outcomes were as follows: graft survival, cardiovascular events, biopsy-proven acute rejection, health-related QOL, and infections. Detailed information on the definition of outcomes is found in Online Resource 2.

### Study risk of bias assessment

We assessed the risk of bias in included studies using ROBINS-I which assesses risk of bias in seven domains: confounding, selection of participants in to the study, classification of interventions, deviations from intended interventions, missing data, measurement of outcomes, and selection of the reported result [17]. Confounding domains and co-interventions are listed in Online Resource 3. Risk of bias assessment was performed independently by two authors (T.A. and N.K.). Disagreements between individual judgments were resolved through consensus-based discussion.

### Effect measures and synthesis methods

Studies reporting adjusted hazard ratio (HR) for mortality and graft loss, the number of cases of each outcome (CVD, acute rejection, and infectious diseases), and the respective mean of each QOL score was integrated into the meta-analyses. Data were combined using the generic inverse-variance method in RevMan 5.4 (Reviewer Manager 5.4, Cochrane, Oxford, UK). Dichotomous outcomes are summarized using pooled HRs with corresponding 95% CI or RRs with 95% CIs. Mean differences with 95% CIs were used as the summary effect measures of continuous outcomes. We visually checked each effect estimate and 95% CI for both individual studies and meta-analysis in a forest plot. If CIs for the results of individual studies had poor overlap, the heterogeneity of intervention effects was evaluated using the *I*^2^ statistic. *I*^2^>50.0% was considered to represent significant heterogeneity. To assess heterogeneity, *I*^2^ was compared between subgroups as described in Online Resource 4. We used a random effects model that incorporated the potential heterogeneity among included studies to synthesize data. Publication bias for each outcome was assessed through visual inspection of the symmetry of the funnel plots. Sensitivity analyses were also performed to evaluate the influence of individual studies on each outcome using a leave-one-out method.

### Certainty assessment

The certainty of evidence was evaluated according to the Grading of Recommendations Assessment, Development and Evaluation (GRADE) criteria, with each outcome being estimated as having a high, moderate, low, or very low level of evidence using the online software, GRADEpro GDT [18–20].

## Results

### Characteristics of included studies

**Figure 1.**
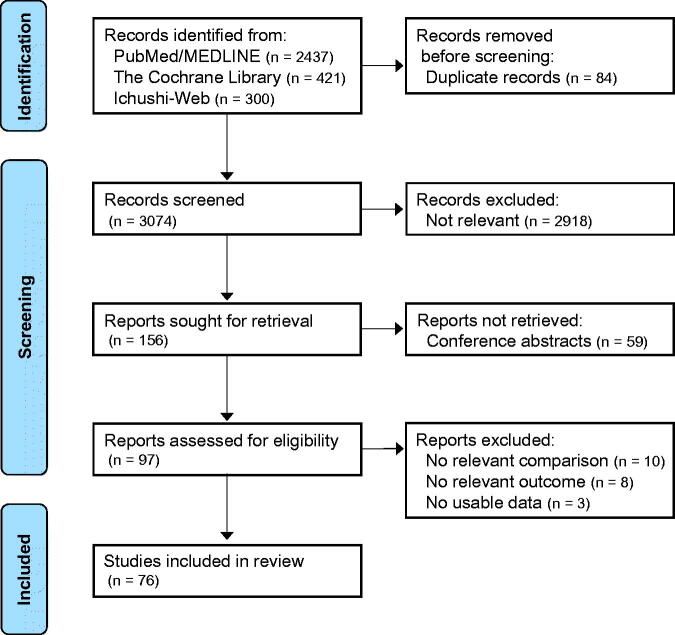
Flow diagram of study inclusion.

After removing duplicated studies, 3074 articles were identified in the initial search. Following screening, 97 articles were fully assessed for eligibility. Of these, 76 observational studies fulfilled the inclusion criteria and were included in the present study (Figure 1). The reviewers agreed on study inclusion for 91.7% of articles (*κ* = 0.81). The main characteristics of the studies are summarized in Table 1 and Supplemental Tables 2–9. Study sample sizes ranged from 23 to 121,853 (median 550), and the enrollment year of study participants ranged from 1968 to 2019.

### Patient survival

Sixty-two studies compared mortality between PEKT patients and non-PEKT patients (sample sizes 44–121,853; median 719.5). Fourteen studies reported the adjusted HR of all-cause mortality, and 10 studies for which detailed information was available were included in the meta-analysis, with a combined sample size of 125,089 individuals (PEKT, 34,846 participants vs. non-PEKT, 90,243 patients).

**Figure 2.**
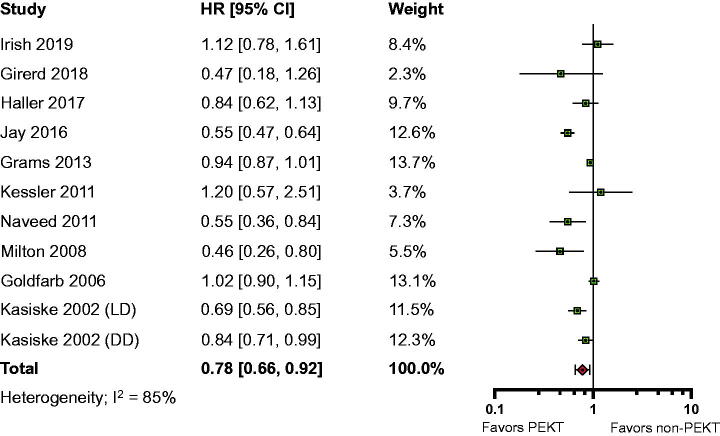
Forest plot of patient mortality.

The overall adjusted HR for all-cause mortality was 0.78 (95% confidence interval [CI], 0.66–0.92; *I*^2^=85%; moderate certainty evidence), indicating that PEKT likely reduced the likelihood of all-cause mortality relative to non-PEKT, with a large heterogeneity (Figure 2). Post hoc subgroup analyses suggested that there was a statistically significant subgroup effect among the dialysis duration subgroup (*p*=.02) (Supplemental Table 10). On the other hand, in the subgroup analysis by donor type, the HRs for all-cause mortality were lower for PEKT, regardless of donor type (Supplemental Fig. 1). In post hoc sensitivity analysis, the leave-one-out analysis showed that no single study had a significant impact on overall estimations (Supplemental Table 11).

Three studies that examined death with functioning graft (DWFG) were included in another meta-analysis, with a combined sample size of 68,912 individuals (PEKT, 32,496 participants vs. non-PEKT, 36,416 patients). The overall adjusted HR for DWFG was 0.74 (95% CI, 0.61–0.89; *I*^2^=82%), indicating that PEKT also likely resulted in a reduction in the likelihood of DWFG relative to non-PEKT (Supplemental Fig. 2).

### Graft survival

**Figure 3.**
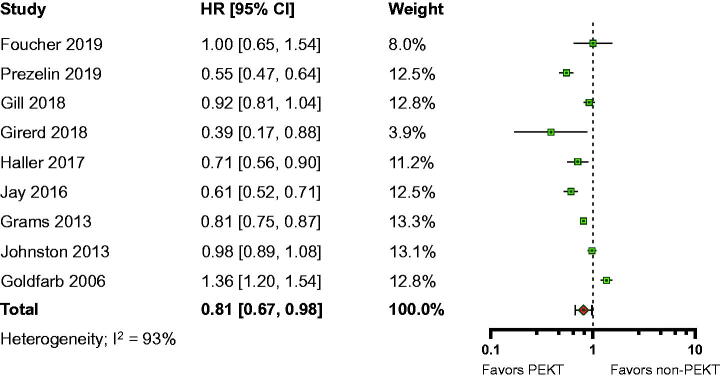
Forest plot of death-censored graft failure.

Sixty-six studies compared the graft survival in PEKT patients with that in non-PEKT patients (sample sizes 44–121,853; median 773). Fourteen studies examined the adjusted HRs for death-censored graft failure (DCGF). Nine studies for which detailed information was available were included in the meta-analysis, with a combined sample size of 142,674 individuals (PEKT, 60,623 participants vs. non-PEKT, 82,051 participants). The overall adjusted HR for DCGF was 0.81 (95% CI, 0.67–0.98; *I*^2^=93%; moderate certainty evidence) (Figure 3). The test for subgroup differences suggested that there was a statistically significant subgroup effect among the publication year subgroups (*p*<.001) (Supplemental Table 10). In post hoc leave-one-out analysis, we found that no single study had a significant impact on the overall estimations (Supplemental Table 11).

### Cardiovascular diseases

In unadjusted analyses, six studies compared the cardiovascular event in PEKT patients with that in non-PEKT patients (sample sizes 44–786; median 155.5). All six studies were included in the meta-analysis, with a combined sample size of 1649 individuals (PEKT, 606 participants vs. non PEKT, 1043 participants). The overall risk ratio (RR) for CVD was 0.90 (95% CI, 0.58–1.40; *I*^2^=0%; very low certainty evidence) (Supplemental Fig. 3). PEKT may have no effect on CVDs, but the evidence is very uncertain.

### Acute rejection

In unadjusted analyses, 39 studies compared acute rejection associated with PEKT with that associated with non-PEKT (sample sizes 23–90,160; median 334). Of these, 19 studies specified that acute rejection was defined as biopsy-proven. Nine studies were included in the meta-analysis, with a combined sample size of 3293 individuals (PEKT, 497 participants vs. non-PEKT, 2796 participants). The overall RR for biopsy-proven acute rejection was 0.75 (95% CI, 0.55–1.03; *I*^2^=36%; very low certainty evidence) (Supplemental Fig. 4). PEKT may have little effect on acute rejection, but the evidence is very uncertain.

### Quality of life

In unadjusted analyses, five studies compared the QOL score in PEKT patients with that in non-PEKT patients (sample sizes 32–1849; median 99). Of these, four studies compared QOL score using the SF-36 (MOS 36-Item Short-Form Health Survey) between PEKT and non-PEKT patients. One study used the AIS (Acceptance of Illness Scale), SWLS (Satisfaction With Life Scale), and STAI (State-Trait Anxiety Inventory) as QOL scores instead [15]. Of the four studies that compared SF-36 scores between PEKT and non-PEKT patients, three studies for which detailed data were available were integrated into the meta-analysis, with a combined sample size of 505 individuals (PEKT, 240 participants vs. non-PEKT, 265). The overall mean differences for each of the eight scaled scores of SF-36 did not differ between PEKT and non-PEKT participants (very low certainty evidence) (Supplemental Fig. 5). For outcomes other than BP (bodily pain) score, there was no evidence of heterogeneity across studies. When the study of Mitsui et al. [21]. was omitted from the *post hoc* analysis, the heterogeneity completely disappeared (*I*^2^ reduced from 67% to 0%). Therefore, PEKT may have no effect on post-transplant QOL scores, but the evidence is very uncertain.

### Infectious diseases

In unadjusted analyses, 10 studies compared the incidence of cytomegalovirus (CMV) infection in PEKT patients with that in non-PEKT patients (sample sizes 23–452; median 84). Nine studies were included in the meta-analysis, with a combined sample size of 1114 individuals (PEKT, 322 participants vs. non-PEKT, 892 participants). The overall RR for CMV infection was 1.04 (95% CI, 0.85–1.29; *I*^2^=0%; very low certainty evidence) (Supplemental Fig. 6). Five studies compared the incidence of urinary tract infection in PEKT patients with that in non-PEKT patients (sample sizes 44–452; median 82). Of these, one study showed that the incidence of urinary tract infections in PEKT patients was significantly lower than that in non-PEKT patients (PEKT 20.3% vs. non-PEKT 44.4%, *p*=.02), but the detailed data that is required for integration into the meta-analysis was not available [17]. The remaining four studies for which detailed data were available were integrated into the meta-analysis, with a combined sample size of 650 individuals (PEKT, 144 participants vs. non-PEKT, 506 participants). The overall RR for urinary tract infection was 0.89 (95% CI, 0.61–1.29; *I*^2^=0%; very low certainty evidence) (Supplemental Fig. 7). Taken together, PEKT may have no effect on CMV infection and urinary tract infection, but the evidence is very uncertain.

### Risk of bias and certainty of evidence

All eligible studies used a cohort design. Risk of bias was assessed in all 76 studies (192 outcomes) and the result is presented in Supplemental Table 12 and Supplemental Figs. 8–13. Funnel plots indicated no substantial publication biases for primary and secondary outcomes (Supplemental Figs. 14–19). A summary of findings in the present study is shown in Table 2.

**Table 2.** Summary of findings.

PEKT compared with non-PEKT in adults with end-stage kidney disease							
Patients: adults with end-stage kidney disease							
Settings: kidney transplantation							
Intervention: PEKT							
Comparison: non-PEKT							
Outcomes	Absolute risk (95% CI)			Relative effect (95% CI)	Number of participants (studies)	Certainty of the evidence (GRADE)	Comments
	Non-PEKT	PEKT	Difference				
Patient mortality	LD			HR 0.78 (0.66–0.92)	125,089 (10 non-randomized studies)	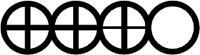 Moderate^b^	
	66 per 1000	52 per 1000 (44–61)^a^					
	DD						
	138 per 1000	109 per 1000 (93–128)^a^					
Death-censored graft failure	LD			HR 0.81 (0.67–0.98)	142,674 (9 non-randomized studies)	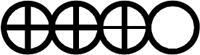 Moderate^c^	
	82 per 1000	67 per 1000 (56–80)^a^					
	DD						
	128 per 1000	105 per 1000 (88–126)^a^					
Cardiovascular disease	55 per 1000	45 per 1000		RR 0.90 (0.58–1.40)	1649 (6 non-randomized studies)	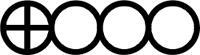 Very low^d^	
Biopsy-proven acute rejection	253 per 1000	155 per 1000		RR 0.75 (0.55–1.03)	3293 (9 non-randomized studies)	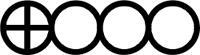 Very low^d^	
Cytomegalovirus infection	268 per 1000	280 per 1000		RR 1.04 (0.85–1.29)	1114 (9 non-randomized studies)	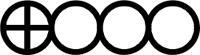 Very low^d^	
Urinary tract infection	128 per 1000	201 per 1000		RR 0.89 (0.61–1.29)	650 (4 non-randomized studies)	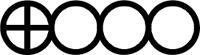 Very low^d^	
Quality of life					(3 non-randomized studies)	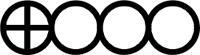 Very low^d^	
PF			0.06 higher scores (3.27 lower to 3.39 higher)		453		
RP			0.86 lower scores (6.38 lower to 4.66 higher)		453		
BP			1.26 lower scores (7.72 lower to 5.19 higher)		443		
GH			0.14 lower scores (3.12 lower to 2.84 higher)		457		
VT			0.38 higher scores (2.84 lower to 3.60 higher)		455		
SF			2.08 lower scores (5.30 lower to 1.13 higher)		450		
RE			0.86 lower scores (7.10 lower to 5.38 higher)		455		
MH			0.75 lower scores (3.53 lower to 2.04 higher)		456		

CI: confidence interval; HR: hazard ratio; RR: relative risk; PF: physical functioning; RP: role physical; BP: bodily pain; GH: general health; VT: vitality; SF: social functioning; RE: role emotional; MH: mental health.

^a^ Baseline risks with 95% confidence intervals were estimated from large, representative observational studies at low risk of bias (unadjusted 5-year survival rates obtained from USRDS 2014 data; available from https://adr.usrds.org/2021).

^b^ Serious unexplained inconsistency (large heterogeneity *I*^2^=85%).

^c^ Serious unexplained inconsistency (large heterogeneity *I*^2^=93%).

^d^ High risk of bias due to confounding and serious imprecision (95% confidence interval includes no effect).

## Discussion

This is the first meta-analysis that compared post-transplant outcomes between PEKT and non-PEKT patients and revealed that PEKT was likely associated with a reduced risk of mortality and graft loss relative to non-PEKT. The reduced mortality risk was probably consistent for different donor sources. However, the incidence of CVD, acute rejection, and infectious disease and post-transplant QOL scores did not significantly differ between PEKT and non-PEKT patients. Taken together, these results suggest that PEKT is the preferred therapeutic approach for adult patients with ESKD, especially in terms of low mortality and graft loss.

Our subgroup analysis of mortality in a living donor transplantation was consistent with ERA-EDTA recommendation for living donor transplantation [11]. In addition, our meta-analysis of deceased donor transplants similarly showed that patient survival was better in PEKT patients than in non-PEKT patients. Therefore, PEKT is the preferred choice for transplantation from both living and deceased donors.

We also conducted post hoc subgroup analyses separated by the duration of dialysis in order to examine the impact of the duration of dialysis before kidney transplantation on post-transplant mortality. The adjusted HR for mortality in PEKT patients vs. short dialysis patients (on dialysis <1.5 years) tended to be lower (0.94, 95% CI, 0.88–1.01). A previous retrospective study that followed transplanted patients for a median of 8.2 years, which was included in the present subgroup meta-analysis, clearly showed, a stepwise dose-dependent increase in mortality with increasing dialysis duration [10]. In contrast, a US dataset clearly indicated that there was no difference in patient mortality between PEKT and non-PEKT patients when the comparison was limited to short-term dialysis patients with a pre-transplant dialysis period of <1 year [22]. Therefore, although we found that the adjusted HR for mortality in PEKT patients vs. short dialysis period patients tended to be lower, the correlation of dialysis duration before transplantation, especially of short-term dialysis, with survival is controversial; therefore, it is difficult to conclude that pre-transplant dialysis duration strongly affects mortality.

The detailed mechanism that leads to a lower mortality in PEKT patients than in non-PEKT patients has not been fully elucidated. Vascular calcification, chronic inflammation, arterial stiffening, and increased risk of infections, which are common in dialysis patients [23–27], may contribute to the higher mortality rate in non-PEKT patients. In fact, the inverse association between eGFR and specific causes of death, including CVD and infection, was reported in a previous study [28]. However, the incidence of CVD itself was not less in PEKT patients in the present study (RR, 0.90; 95% CI, 0.58–1.40; *I*^2^=0%; very low certainty evidence), indicating that PEKT may not be superior to non-PEKT in terms of CVD prevention. However, it should be noted that the incidence rate of CVD in studies included our meta-analyses was lower than that in a previous large database study [29]. In addition, infectious disease is also an important factor that contributes to mortality in kidney transplant recipients. The present study found that there were no significant differences in the risk of developing CMV infection or urinary tract infection between PEKT and non-PEKT patients, although viral infection and urinary tract infection only cause a small number of deaths in kidney transplant patients [30]. Furthermore, previous studies have shown that there is no significant difference in the proportion of deaths caused by infectious diseases between PEKT and non-PEKT patients [10,31]. Therefore, because there is no clear evidence that PEKT reduces CVD or infections, the present study did not elucidate the reasons that could explain the lower mortality with PEKT.

In terms of graft survival, PEKT also showed a benefit over non-PEKT. A French database study clearly indicated the gradual increase in HR for graft failure with pre-transplant dialysis duration [32]. However, another cohort study indicated the advantage of PEKT on graft survival but did not find the significant association between rate of graft loss and pre-transplant dialysis duration [10]. No plausible reason for this discrepancy has yet been found, but a national cohort study in the United States demonstrated that pre-listing ESKD time had a stronger impact on graft loss than waiting time on the deceased donor transplant list, and that pre-listing ESKD time was associated with comorbid conditions, socioeconomic status, and access to healthcare [33], indicating the possibility that these factors, not pre-transplant dialysis time, may directly affect graft loss. Developing acute rejection can be considered another factor that strongly affects graft survival [34]. The initiation of dialysis treatment is known to improve T-cell activation in ESKD patients [35], although it is not known whether this causes acute rejection. Therefore, pre-transplant dialysis may increase the risk of acute rejection. In the present study, the overall RR for biopsy-proven acute rejection tended to be lower (RR, 0.75; 95% CI, 0.55–1.03; *I*^2^=36%; very low certainty evidence). Less rejection may partly be the reason for the lower HR for graft failure in PEKT patients, but because the result regarding acute rejection had very low certainty evidence, we cannot conclude a causal relationship between graft failure and rejection in the present study.

For patients on dialysis, receiving a kidney transplant can lead to independence from dialysis and improve their QOL [36]. The QOL scores before transplantation in PEKT patients was better than that of dialysis patients [19] because they have not experienced dialysis therapy. However, PEKT can also improve QOL and mental satisfaction after transplantation, albeit slowly [21]. As the pre-transplant QOL scores themselves are different between PEKT and non-PEKT patients, it is difficult to directly compare the improvement in QOL scores before and after transplant between both groups. Therefore, we simply compared their QOL scores after transplantation, and found no significant difference between them.

The strengths of this systematic review include a comprehensive review of qualitative and quantitative study evidence, risk of bias assessment using ROBINS-I (Risk of Bias In Non-randomized Studies – of Interventions), and grading the certainty of evidence following the GRADE approach. However, these strengths should be balanced against the high heterogeneity across studies in the primary outcome. Post hoc subgroup analyses did not explain the heterogeneity in patient mortality across studies. However, among the dialysis duration subgroups, the test for subgroup differences suggested that there was a statistically significant subgroup effect, possibly and partially due to the small number of studies. Therefore, we judged that there was serious inconsistency in the primary outcome between studies.

Some limitations should be noted in the present meta-analyses. First, the time difference in ESKD period between PEKT and non-PEKT patients (i.e., lead time) may have affected the clinical advantage of PEKT. A previous registry study suggested that accounting for lead time moved estimates toward a survival disadvantage for PEKT, although this was in comparison to those transplanted within 6 months of commencing dialysis [37]. However, it is difficult to accurately assess lead-time bias when the duration of dialysis is so short, as the prognostic difference by lead-time is unlikely to be clear. Although the issue of lead-time bias was not resolved in the present meta-analysis, future clinical studies should be designed to eliminate lead-time bias in order to assess the ‘true’ clinical efficacy of PEKT. If an RCT could be conducted in ESKD patients randomly assigning PEKT vs. non-PEKT as an intervention, it would be possible to assess the ‘true’ clinical efficacy of PEKT, completely removing the influence of lead time, but it is ethically difficult to implement. Alternatively, it may be possible to reduce the influence of lead time by conducting a retrospective study that observes the prognosis of ESKD patients in the PEKT and non-PEKT groups from the pre-transplant time point of matching eGFR in both groups. Second, because we integrated unadjusted RRs for CVD, acute rejection, and infections and unadjusted mean differences for each QOL score in PEKT patients vs. non-PEKT patients in our meta-analyses, baseline confounding was not fully eliminated; therefore, bias due to confounding may have affected the results (Supplemental Table 11). Third, no study evaluated CVD, acute rejection, or infectious diseases as a primary outcome; all have been evaluated as secondary outcomes or components thereof. Fourth, there are some variations in the definitions of CVD and infectious diseases among studies. Although we extracted myocardial infarction, stroke, CMV infection, and urinary tract infection as outcomes, their diagnostic methods were not described or defined in detail in each study; therefore, our meta-analyses may not have been able to assess identical outcomes.

To our knowledge, this is the first meta-analysis that examines the post-transplant outcome of PEKT and non-PEKT. The present meta-analysis shows the potential benefits of PEKT, especially regarding patient and graft survival. Nevertheless, the risk of rejection and infection in PEKT patients was comparable to that in non-PEKT patients, indicating that PEKT may not lead to any disadvantage. On the basis of these findings, we recommend that transplantation is performed pre-emptively.

## Supplementary Material

Supplemental Material

Supplemental Material

## Data Availability

The data used to support the findings of this study are included in the main text or supplementary materials. Any remaining information are available from the corresponding author upon reasonable request.
