# Supplementary material for: Efficacy of pre-emptive kidney transplantation for adults with end-stage kidney disease: a systematic review and meta-analysis
Source: Ren Fail. 2023 Jan 27;45(1):2169618. doi: 10.1080/0886022X.2023.2169618 (PMC9888453; doi:10.1080/0886022X.2023.2169618)
Supplement: Supplemental Material [file IRNF_A_2169618_SM2285.pdf]

Supplemental Item 1. List of items included in the data extraction

(1) Population: country, sample size, baseline characteristics of participants (age, sex, ethnicity, comorbidities, donor source, smoking status, etiology of ESKD, number of HLA mismatches, ABO blood group compatibility), and donor information (source [living/deceased], age, extended criteria donor status, panel reactive antibody)

(2) Intervention/exposure: PEKT definition

(3) Comparison: dialysis duration

(4) Outcome: definition of mortality (death with/without functioning graft), graft loss (death censored or not), cardiovascular disease, acute rejection (biopsy-proven or not), infectious diseases, and QOL (which scores were used for quantification of QOL)

(5) Studies: publication year, study design

Information Classification: General

Supplemental Item 2. Definition of outcomes

Primary outcome: patient survival

At the time of study design, we planned a priori to evaluate patient survival as (1) the time from kidney transplantation to death or (2) death from any cause during the trial follow-up period and at 1, 3, 5, and 10 years after transplantation as the primary outcome (recorded on PROSPERO [ID: CRD42021269163]). Unfortunately, the follow-up period was different in each study and in each patient; therefore, there was difficulty in synthesizing the results. To address this problem, we decided to integrate only studies that reported adjusted hazard ratios (HRs) for all-cause mortality to evaluate patient survival.

Secondary outcome: graft survival, cardiovascular events, biopsy-proven acute rejection, health related quality of life (QOL), and infections (bacterial or viral infections)

At the time of study design, we planned a priori to evaluate graft survival as (1) the time from kidney transplantation to graft failure or (2) death-censored graft survival/death with functioning graft during the trial follow-up period and at 1, 3, 5, and 10 years after transplantation (recorded on PROSPERO [ID: CRD42021269163]). For the same reason as the primary outcome, we decided to integrate only studies that reported adjusted HRs for death-censored graft failure to evaluate graft survival. We also decided to integrate studies that reported adjusted HRs for death with functioning graft.
Moreover, we planned a priori to define cardiovascular events as "cardiovascular death, myocardial infarction, or ischemic stroke." However, since there were no studies independently reporting the number of cardiovascular death events as an outcome of CVD, we decided to evaluate the risk ratios (RRs) of "myocardial infarction or ischemic stroke" as the CVD outcome in the present study.

Information Classification: General

Supplemental Item 3. List of confounding domains and co-interventions

(1) Patient mortality

Confounding domains: age, sex, smoking status, hypertension, diabetes, obesity, prior cardiovascular diseases, cancer, etiology of ESKD

Co-interventions: immunosuppressants, general conditions during transplant surgery

(2) Graft survival

Confounding domains: recipient/donor age, number of HLA mismatches, donor sources, extended criteria donor, panel reactive antibody, etiology of ESKD, ABO blood group

Co-interventions: immunosuppressants, general condition during transplant surgery

(3) Cardiovascular disease

Confounding domains: age, sex, smoking, hypertension, diabetes, obesity, prior cardiovascular diseases, etiology of ESKD

Co-interventions: immunosuppressants, general condition during transplant surgery

(4) Acute rejection

Confounding domains: recipient/donor age, number of HLA mismatches, donor sources, extended criteria donor, panel reactive antibody, ABO blood group

Co-interventions: immunosuppressants, general condition during transplant surgery

(5) Quality of life

Confounding domains: age, sex, smoking status, hypertension, diabetes, obesity, prior cardiovascular diseases, cancer, etiology of ESKD

Co-interventions: immunosuppressants, general condition during transplant surgery

(6) Infectious diseases

Confounding domains: age, sex, smoking status, hypertension, diabetes, obesity, prior cardiovascular diseases, etiology of ESKD

Co-interventions: immunosuppressants, general condition during transplant surgery

Information Classification: General

Supplemental Item 4. Subgroup analysis methods

To assess the heterogeneity of the clinical effects of PEKT, the following subgroup analyses were planned to be performed in the pre-specification (recorded on PROSPERO [ID: CRD42021269163]).

(1) sex (male/female)
(2) age (<65 years/≥65 years)
(3) dialysis (<1 year/1–5 years/≥5 years).

However, since there were no studies evaluating adjusted hazard ratios for patient mortality and graft loss by sex, age, or duration of dialysis as described above, we changed the definition of subgroups as follows and conducted the subgroup analyses.

(1) Patient location (North America, South America, Oceania, Asia, Europe, and Africa)
(2) Year of publication (2000 and earlier, 2001–2010, and 2011 and later)
(3) Donor source (living donor, deceased donor, mixed/unknown)
(4) Dialysis duration (<1.5 years or not)
(5) Primary transplantation/repeat transplantation

Information Classification: General

Supplemental Item 5. Reference list

1. Auneau-Enjalbert L, et al. Investigation of measurement invariance in longitudinal health-related quality of life in preemptive or previously dialyzed kidney transplant recipients. Qual Life Res. 2021.
2. Aytekin S, et al. Perioperative Comparison of Preemptive and Non-Preemptive Renal Transplant Recipients. Turk J Anaesthesiol Reanim. 2020;48(2):102-7.
3. Mitsui Y, et al. Quality of Life and Mental Satisfaction Improve Slowly in Preemptive Kidney Transplantation Compared With Nonpreemptive Kidney Transplantation. Transplant Proc. 2020;52(3):740-7.
4. Franco A, et al. Pre-emptive deceased-donor kidney transplant: A matched cohort study. Nefrologia (Engl Ed). 2020;40(1):32-7.
5. Irish GL, et al. Quantifying lead time bias when estimating patient survival in preemptive living kidney donor transplantation. Am J Transplant. 2019;19(12):3367-76.
6. Kim HY, et al. Comparison of Clinical Outcomes Between Preemptive Transplant and Transplant After a Short Period of Dialysis in Living-Donor Kidney Transplantation: A Propensity-Score-Based Analysis. Ann Transplant. 2019;24:75-83.
7. Foucher Y, et al. Lack of impact of pre-emptive deceased-donor kidney transplantation on graft outcomes: a propensity score-based study. Nephrol Dial Transplant. 2019;34(5):886-91.
8. Prezelin-Reydit M, et al. Prolonged dialysis duration is associated with graft failure and mortality after kidney transplantation: results from the French transplant database. Nephrol Dial Transplant. 2019;34(3):538-45.
9. Mochizuki T, et al. Clinical research of preemptive kidney transplantation. Jin to Toseki (Kidney and Dialysis). 2019;86 Suppl:72-4. (Japanese).
10. Matsumura S, et al. Comparative Study on Variation of Quality of Life of Patients of Preemptive Kidney Transplantation and Nonpreemptive Kidney Transplantation. Transplant Proc. 2018;50(10):3321-8.
11. Aufhauser DD, et al. Impact of prolonged dialysis prior to renal transplantation. Clin Transplant. 2018;32(6):e13260.
12. Gill JS, et al. Variation in Dialysis Exposure Prior to Nonpreemptive Living Donor Kidney Transplantation in the United States and Its Association With Allograft Outcomes. Am J Kidney Dis. 2018;71(5):636-47.
13. Mursawa H, et al. Slow Progression of Aortic Calcification Is a Potential Benefit of Pre-emptive Kidney Transplantation. Transplant Proc. 2018;50(1):145-9.
14. Girerd S, et al. Preemptive second kidney transplantation is associated with better graft

survival compared with non-preemptive second transplantation: a multicenter French 2000-2014 cohort study. Transpl Int. 2018;31(4):408-23.

15. Haller MC, et al. Dialysis Vintage and Outcomes after Kidney Transplantation: A Retrospective Cohort Study. Clin J Am Soc Nephrol. 2017;12(1):122-30.
16. Okumi M, et al. Preemptive kidney transplantation: a propensity score matched cohort study. Clin Exp Nephrol. 2017;21(6):1105-12.
17. Nakagawa Y, et al. Preemptive kidney transplantation. Jin to Toseki (Kidney and Dialysis). 2017;83:162-7. (Japanese).
18. Gadelkareem RA, et al. Living donor kidney transplantation in the hemodialysis-naive and the hemodialysis-exposed: A short term prospective comparative study. Afr J Urol. 2017;23(1):56-61.
19. Girerd S, et al. Temporal Trend and Time-Varying Effect of Preemptive Second Kidney Transplantation on Graft Survival: A 30-Year Single-Center Cohort Study. Transplant Proc. 2016;48(8):2663-8.
20. Bzoma B, et al. Psychological Well-Being in Patients After Preemptive Kidney Transplantation. Transplant Proc. 2016;48(5):1515-8.
21. Goto N, et al. Association of Dialysis Duration with Outcomes after Transplantation in a Japanese Cohort. Clin J Am Soc Nephrol. 2016;11(3):497-504.
22. Jay CL, et al. Reassessing Preemptive Kidney Transplantation in the United States: Are We Making Progress? Transplantation. 2016;100(5):1120-7.
23. Noda T, et al. Comparison of preemptive kidney transplantation with non-preemptive kidney transplantation in a single center. Nishinihon Hinyoukika (The Nishinihon Journal of Urology). 2016;78:499-502. (Japanese).
24. Florit EA, et al. Pre-Emptive Retransplantation in Patients With Chronic Kidney Graft Failure. Transplant Proc. 2015;47(8):2351-3.
25. Morales E, et al. Preemptive kidney transplantation in elderly recipients with kidneys discarded of very old donors: A good alternative. Nefrologia. 2015;35(3):246-55.
26. Unsal MG, et al. Comparison of Preemptive Kidney Transplantation With Nonpreemptive Kidney Transplantation in a Single Center: A Follow-up Study. Transplant Proc. 2015;47(5):1385-7.
27. Nakamura T, et al. Advantages and disadvantages of pre-emptive kidney transplantation: results from a single transplantation center. Transplant Proc. 2015;47(3):626-9.
28. Oishi K, et al. An evaluation of preemptive kidney transplantation. Konnichi no Ishoku (Transplantation now). 2015;28:225-8. (Japanese).
29. Debska-Slizien A, et al. Preemptive kidney transplantation: analysis of kidney grafts from

Information Classification: General

the same donor. Transplant Proc. 2014;46(8):2654-9.
30. Kohei N, et al. Influence of dialysis duration on the outcome of living kidney transplantation. Ther Apher Dial. 2014;18(5):481-8.
31. Ryosaka M, et al. Comparison of patient and graft survivalsbetween renal post-dialysis kidney transplant recipients and preemptive kidney transplant recipients. Nihon Rinsho Jin Ishoku Gakkai Zasshi (Journal of Japanese Society for Clinical Renal Transplantation). 2014;2:199-202. (Japanese).
32. Sayin B, et al. Comparison of preemptive kidney transplant recipients with nonpreemptive kidney recipients in single center: 5 years of follow-up. Int J Nephrol Renovasc Dis. 2013;6:95-9.
33. Bozkurt B, et al. Patient and graft survival after pre-emptive versus non-pre-emptive kidney transplantation: a single-center experience from Turkey. Transplant Proc. 2013;45(3):932-4.
34. Johnston O, et al. Risks and benefits of preemptive second kidney transplantation. Transplantation. 2013;95(5):705-10.
35. Grams ME, et al. Preemptive deceased donor kidney transplantation: considerations of equity and utility. Clin J Am Soc Nephrol. 2013;8(4):575-82.
36. Hayashida Y, et al. Effect of duration of dialysis therapy on the outcome of living kidney transplantation. Nihon Rinsho Jin Ishoku Gakkai Zasshi (Journal of Japanese Society for Clinical Renal Transplantation). 2013;1:50-4. (Japanese).
37. Luo M, et al. Preemptive deceased-donor renal transplant in adults: single-center experience and outcome. Exp Clin Transplant. 2012;10(2):101-4.
38. Keith DS. Preemptive deceased donor kidney transplant not associated with patient survival benefit in minority kidney transplant recipients. Clin Transplant. 2012;26(1):82-6.
39. Naveed A, et al B. Preemptive kidney transplantation in systemic lupus erythematosus. Transplant Proc. 2011;43(10):3713-4.
40. Rigo DH, et al. Preemptive kidney transplantation: experience in two centers. Transplant Proc. 2011;43(9):3355-8.
41. Kessler M, et al. Does pre-emptive kidney transplantation with a deceased donor improve outcomes? Results from a French transplant network. Transpl Int. 2011;24(3):266-75.
42. Son YK, et al. Clinical outcome of preemptive kidney transplantation in patients with diabetes mellitus. Transplant Proc. 2010;42(9):3497-502.
43. Jung GO, et al. Can preemptive kidney transplantation guarantee longer graft survival in living-donor kidney transplantation? Single-center study. Transplant Proc.

Information Classification: General

2010;42(3):766-74.
44. Witczak BJ, et al. Experience from an active preemptive kidney transplantation program--809 cases revisited. Transplantation. 2009;88(5):672-7.
45. Salvadori M, et al. Preemptive cadaveric renal transplantation: fairness and utility in the case of high donation rate-pilot experience of Tuscany region. Transplant Proc. 2009;41(4):1084-6.
46. Yoo SW, et al. Preemptive living-donor renal transplantation: outcome and clinical advantages. Transplant Proc. 2009;41(1):117-20.
47. Milton CA, et al. Pre-emptive renal transplantation from living donors in Australia: effect on allograft and patient survival. Nephrology (Carlton). 2008;13(6):535-40.
48. Ishikawa N, et al. Preemptive kidney transplantation of living related or unrelated donor-recipient combinations. Transplant Proc. 2008;40(7):2294-6.
49. Joo KW, et al. Preemptive transplantation and long-term outcome in living donor kidney transplantation, single-center experience. Transplant Proc. 2007;39(10):3061-4.
50. Pour-Reza-Gholi F, et al. Is preemptive kidney transplantation preferred? Updated study. Urol J. 2007;4(3):155-8.
51. Perez-Flores I, et al. Preemptive kidney transplant from deceased donors: an advantage in relation to reduced waiting list. Transplant Proc. 2007;39(7):2123-4.
52. Innocenti GR, et al. Preemptive living donor kidney transplantation: do the benefits extend to all recipients? Transplantation. 2007;83(2):144-9.
53. Kennedy SE, et al. Waiting time and outcome of kidney transplantation in adolescents. Transplantation. 2006;82(8):1046-50.
54. Debska-Slizien A, et al. A single center experience in preemptive kidney transplantation. Transplant Proc. 2006;38(1):49-52.
55. Goldfarb-Rumyantzev AS, et al. The role of pre-emptive re-transplant in graft and recipient outcome. Nephrol Dial Transplant. 2006;21(5):1355-64.
56. Becker BN, et al. Preemptive transplantation for patients with diabetes-related kidney disease. Arch Intern Med. 2006;166(1):44-8.
57. Abou Ayache R, et al. Preemptive renal transplantation in adults. Transplant Proc. 2005;37(6):2817-8.
58. Goldfarb-Rumyantzev A, et al. Duration of end-stage renal disease and kidney transplant outcome. Nephrol Dial Transplant. 2005;20(1):167-75.
59. Gill JS, et al. Why do preemptive kidney transplant recipients have an allograft survival advantage? Transplantation. 2004;78(6):873-9.
60. el-Agroudy AE, et al. Preemptive living-donor kidney transplantation: clinical course and outcome. Transplantation. 2004;77(9):1366-70.

Information Classification: General

61. Simforoosh N, et al. Is preemptive renal transplantation preferred? Transplant Proc. 2003;35(7):2598-601.
62. Mange KC, et al. Dialysis prior to living donor kidney transplantation and rates of acute rejection. Nephrol Dial Transplant. 2003;18(1):172-7.
63. Nishikawa K, et al. Outcome of preemptive renal transplantation versus waiting time on dialysis. Clin Transpl. 2002:367-77.
64. Meier-Kriesche HU, et al. Waiting time on dialysis as the strongest modifiable risk factor for renal transplant outcomes: a paired donor kidney analysis. Transplantation. 2002;74(10):1377-81.
65. Kasiske BL, et al. Preemptive kidney transplantation: the advantage and the advantaged. J Am Soc Nephrol. 2002;13(5):1358-64.
66. Mange KC, et al. Effect of the use or nonuse of long-term dialysis on the subsequent survival of renal transplants from living donors. N Engl J Med. 2001;344(10):726-31.
67. Meier-Kriesche HU, et al. Effect of waiting time on renal transplant outcome. Kidney Int. 2000;58(3):1311-7.
68. Papalois VE, et al. Pre-emptive transplants for patients with renal failure: an argument against waiting until dialysis. Transplantation. 2000;70(4):625-31.
69. John AG, et al. Preemptive live-related renal transplantation. Transplantation. 1998;66(2):204-9.
70. Asderakis A, et al. Pre-emptive kidney transplantation: the attractive alternative. Nephrol Dial Transplant. 1998;13(7):1799-803.
71. Roake JA, et al. Preemptive cadaveric renal transplantation--clinical outcome. Transplantation. 1996;62(10):1411-6.
72. Berthoux FC, et al. Transplantation Report. 2: Pre-emptive renal transplantation in adults aged over 15 years. The EDTA-ERA Registry. European Dialysis and Transplant Association-European Renal Association. Nephrol Dial Transplant. 1996;11 Suppl 1:41-3.
73. Ekstrand A. Kidney transplantation with and without prior dialysis therapy in diabetic patients with end-stage renal failure. Scand J Urol Nephrol. 1993;27(1):83-7.
74. Cacciarelli TV, et al. The influence of mode of dialysis pretransplantation on long-term renal allograft outcome. Ren Fail. 1993;15(4):545-50.
75. Katz SM, et al. Preemptive transplantation--an analysis of benefits and hazards in 85 cases. Transplantation. 1991;51(2):351-5.
76. Migliori RJ, et al. Renal transplantation done safely without prior chronic dialysis therapy. Transplantation. 1987;43(1):51-5.

Information Classification: General

Supplemental Table 1. Search strategy for PubMed/MEDLINE and The Cochrane Library databases

Database	Search strategies
PubMed/MEDLINE	(“kidney transplantation"[MeSH Terms] OR “renal transplantation”[tiab] OR “renal transplantations”[tiab] OR ((transplantation[MeSH Terms] OR "transplantation"[All Fields] OR "transplantations"[All Fields]) AND renal[tiab]) OR “transplantation, renal”[tiab] OR (("transplantation"[Subheading] OR "transplantation"[All Fields] OR "grafting"[All Fields] OR "transplantation"[MeSH Terms]) AND kidney[tiab]) OR “kidney grafting”[tiab] OR “transplantation, kidney”[tiab] OR “kidney transplantations”[tiab] OR (("transplantation"[MeSH Terms] OR "transplantation"[All Fields] OR "transplantations"[All Fields]) AND kidney[tiab]) OR “kidney transplant recipient”[tiab] OR “kidney transplant recipients”[tiab]) AND ((pre-emptive[MeSH Terms] OR preemptive[tiab] OR (prior[All Fields] AND dialysis[All Fields]) OR (before[All Fields] AND dialysis[All Fields]) OR pre-emptive[All Fields] OR pre emptive[All Fields]))
The Cochrane Library	(kidney transplantation OR kidney transplantions OR renal transplantation OR renal transplantions OR kidney grafting) AND (pre emptive OR pre-emptive OR preemptive OR prior to dialysis OR before initiation dialysis)

Information Classification: General

Supplemental Table 2. Outcomes of studies included in the systematic review

Study	Outcomes					
	Patient Survival	Graft Survival	CVD	Acute Rejection	QOL	Infectious Disease
Auneau-Enjalbert [1], 2021					●	
Aytekin [2], 2020	●			●		
Mitsui [3], 2020					●	
Franco [4], 2020	●	●		●		
Irish [5], 2019	●					
Kim [6], 2019	●	●				
Foucher [7], 2019		●				
Prezelin-Reydit [8], 2019	●	●				
Mochizuki [9], 2019		●		●		
Matsumura [10], 2018					●	
Aufhauser [11], 2018	●	●				
Gill [12], 2018	●	●				

Information Classification: General

Mursawa [13], 2018		●				
Girerd [14], 2018	●	●		●		
Haller [15], 2017	●	●				
Okumi [16], 2017	●	●	●			●
Nakagawa [17], 2017	●	●				
Gadelkareem [18], 2017	●	●		●		
Girerd [19], 2016		●				
Bzoma [20], 2016					●	
Goto [21], 2016	●	●	●			
Jay [22], 2016	●	●				
Noda [23], 2016				●		●
Florit [24], 2015	●	●		●		
Morales [25], 2015	●	●		●		● ○

Information Classification: General

Unsal [26], 2015	●	●		●	
Nakamura [27], 2015	●	●		●	● ○ ■
Oishi [28], 2015	●			●	●
Dębska-Ślizień [29], 2014	●	●		●	● ○
Kohei [30], 2014	●	●		●	
Ryosaka [31], 2014	●	●			
Sayin [32], 2013	●	●		●	■
Bozkurt [33], 2013	●	●			
Johnston [34], 2013	●	●		●	
Grams [35], 2013	●	●			
Hayashida [36], 2013		●	●	●	● ■
Luo [37], 2012	●	●		●	
Keith [38], 2012	●	●			

Information Classification: General

Naveed [39], 2011	●	●			
Rigo [40], 2011				●	
Kessler [41], 2011	●	●		●	
Son [42], 2010	●	●	●	●	■
Jung [43], 2010	●	●		●	● ○ ■
Witczak [44], 2009	●	●			
Salvadori [45], 2009	●	●			
Yoo [46], 2009	●	●		●	
Milton [47], 2008	●	●		●	
Ishikawa [48], 2008	●	●		●	● ○
Joo [49], 2007	●	●		●	■
Pour-Reza-Gholi [50], 2007	●	●		●	
Pérez-Flores [51], 2007	●	●		●	

Information Classification: General

Innocenti [52], 2007	●	●	●	●	■
Kennedy [53], 2006		●			
Dębska-Ślizień [54], 2006	●	●		●	
Goldfarb-Rumyantzev [55], 2006	●	●			
Becker [56], 2006	●	●			
Abou Ayache [57], 2005	●	●			
Goldfarb-Rumyantzev [58], 2005	●	●			
Gill [59], 2004	●	●		●	
el-Agroudy [60], 2004	●	●		●	■
Simforoosh [61], 2003	●	●		●	
Mange [62], 2003				●	
Nishikawa [63], 2002	●	●		●	
Meier-Kriesche [64], 2002		●			

Information Classification: General

Kasiske [65], 2002	●	●				
Mange [66], 2001	●	●				
Meier-Kriesche [67], 2000	●	●				
Papalois [68], 2000	●	●		●	●	
John [69], 1998	●	●				
Asderakis [70], 1998	●	●		●		
Roake [71], 1996	●	●				
Berthoux [72], 1996	●	●				
Ekstrand [73], 1993	●	●	●	●		● ■
Cacciarelli [74], 1993	●	●		●		
Katz [75], 1991	●	●		●		
Migliori [76], 1987	●	●				

CVD, cardiovascular disease; QOL, quality of life

In the Infection column, blue circles indicate cytomegalovirus infection, white circles indicate urinary tract infection, and blue squares indicate other infectious diseases.

Information Classification: General

References are listed in Online Resource 5.

Information Classification: General

Supplemental Table 3. Overview of studies included in the meta-analysis of patient mortality

Author	Year	PEKT Total No.	Non-PEKT Total No.	Adjusted HR (95% CI)
Irish [5]	2019	699	699	1.12 (0.78–1.61)
Girerd [14]	2018	93	1213	0.47 (0.18–1.26)
Haller [15]	2017	406	1814	0.84 (0.62–1.13)
Jay [22]	2016	14503	17503	0.55 (0.47–0.64)
Grams [35]	2013	10992	14428	0.94 (0.87–1.01)
Naveed [39]	2011	730	7271	0.55 (0.36–0.84)
Kessler [41]	2011	118	1467	1.20 (0.57–2.51)
Milton [47]	2008	578	2025	0.46 (0.26–0.80)
Goldfarb-Rumyantzev [55]	2006	1609	10105	1.02 (0.90–1.15)
Kasiske [LD] [65]	2002	3141	9937	0.69 (0.56–0.85)
Kasiske [DD] [65]	2002	1977	23781	0.84 (0.71–0.99)

References are listed in Online Resource 5.

Information Classification: General

Supplemental Table 4. Overview of studies included in the meta-analysis of graft survival

Author	Year	PEKT Total No.	Non-PEKT Total No.	Adjusted HR (95% CI)
Foucher [7]	2019	500	490	1.00 (0.65–1.54)
Prezelin-Reydit [8]	2019	2770	18183	0.55 (0.47–0.64)
Gill [12]	2018	26217	4158	0.92 (0.81–1.04)
Girerd [14]	2018	93	1213	0.39 (0.17–0.88)
Haller [15]	2017	430	1896	0.71(0.56–0.90)
Jay [22]	2016	14503	17503	0.61 (0.52–0.71)
Grams [35]	2013	10992	14428	0.81 (0.75–0.87)
Johnston [34]	2013	3509	14075	0.98 (0.89–1.08)
Goldfarb-Rumyantzev [55]	2006	1609	10105	1.36 (1.20–1.54)

References are listed in Online Resource 5.

Information Classification: General

Supplemental Table 5. Overview of studies included in the meta-analysis of cardiovascular disease

		PEKT			Non-PEKT			
**Author**	**Year**	**No.**	**Total No.**	**%**	**No.**	**Total No.**	**%**	**Risk Ratio (95% CI)**
Okumi [16]	2017	3	93	3.2	5	93	5.4	0.60 (0.15–2.44)
Goto [21]	2016	2	239	0.8	6	547	1.1	0.76 (0.16–3.75)
Hayashida [36]	2013	0	29	0.0	1	15	6.7	0.18 (0.01–4.12)
Son [42]	2010	2	30	6.7	6	40	15.0	0.44 (0.10–2.05)
Innocenti [52]	2007	14	191	7.3	17	247	6.9	1.06 (0.54–2.11)
Ekstrand [73]	1993	6	24	25.0	22	101	21.8	1.15 (0.52–2.52)

References are listed in Online Resource 5.

Information Classification: General

Supplemental Table 6. Overview of studies included in the meta-analysis of biopsy-proven acute rejection

		PEKT			Non-PEKT			
Author	Year	No.	Total No.	%	No.	Total No.	%	Risk Ratio (95% CI)
Girerd [14]	2018	7	93	7.5	229	1221	18.8	0.40 (0.19–0.83)
Morales [25]	2015	6	26	23.1	1	26	3.8	6.00 (0.78–46.42)
Kohei [30]	2014	8	21	38.1	263	638	41.2	0.92 (0.53–1.61)
Sayin [32]	2013	12	37	32.4	28	63	44.4	0.73 (0.42–1.25)
Luo [37]	2012	4	32	12.5	43	132	32.6	0.38 (0.15–0.99)
Son [42]	2010	3	30	10.0	5	40	12.5	0.80 (0.21–3.09)
Jung [43]	2010	16	62	25.8	92	390	23.6	1.09 (0.69–1.73)
Ishikawa [48]	2008	1	5	20.0	8	39	20.5	0.97 (0.15–6.26)
Innocenti [52]	2007	20	191	10.5	39	247	15.8	0.66 (0.40–1.10)

References are listed in Online Resource 5.

Information Classification: General

Supplemental Table 7. Overview of studies included in the meta-analysis of SF-36 scores

			PEKT			Non-PEKT			
**Author**	**Year**	**Score**	**Patient No.**	**Mean**	**SD**	**Patient No.**	**Mean**	**SD**	**Mean Difference (95% CI)**
Auneau-Enjalbert [1]	2021	PF	148	77.6	24.3	174	76.2	22.4	1.44 (−3.70, 6.58)
		RP	149	57.1	39.2	173	51.7	44.2	5.32 (−3.79, 14.43)
		BP	144	70.8	23.3	168	67.4	24.0	3.36 (−1.89, 8.61)
		GH	151	58.6	18.0	175	57.2	19.9	1.41 (−2.71, 5.53)
		VT	151	58.6	18.8	173	57.2	20.5	1.36 (−2.93, 5.65)
		SF	148	75.9	23.4	171	76.7	20.6	−0.75 (−5.62, 4.12)
		RE	150	69.1	39.8	174	63.7	42.6	5.41 (−3.56, 14.38)
		MH	152	72.1	17.5	173	71.2	16.8	0.88 (−2.86, 4.62)
Mitsui [3]	2020	PF	12	49.7	9.8	20	50.9	7.5	−1.20 (−7.65, 5.25)
		RP	12	48.2	8.1	20	50.7	9.2	−2.50 (−8.60, 3.60)
		BP	12	50.5	10.5	20	57.5	5.4	−7.00 (−13.39, −0.61)
		GH	12	47.2	8.0	20	48.8	7.2	−1.60 (−7.12, 3.92)
		VT	12	53.2	7.8	20	53.0	8.8	0.20 (−5.66, 6.06)
		SF	12	49.9	7.2	20	52.2	7.3	−2.30 (−7.48, 2.88)
		RE	12	50.0	7.5	20	52.3	8.8	−2.30 (−8.03, 3.43)
		MH	12	53.8	6.9	20	55.0	8.9	−1.20 (−6.72, 4.32)
Matsumura [10]	2018	PF	50	87.5	15.1	49	88.2	15.1	−0.70 (−6.65, 5.25)
		RP	50	81.5	27.6	49	86.7	21.8	−5.20 (−15.96, 5.56)
		BP	50	82.7	19.0	49	83.3	21.0	−0.60 (−8.49, 7.29)
		GH	50	54.9	18.2	49	57.1	16.8	−2.20 (−9.10, 4.70)
		VT	50	65.1	24.0	49	68.4	20.2	−3.30 (−12.03, 5.43)
		SF	50	84.0	21.8	49	88.9	16.7	−4.90 (−12.54, 2.74)
		RE	50	78.3	33.1	49	85.9	32.9	−7.60 (−20.60, 5.40)

Information Classification: General

	MH	50	75.0	18.1	49	79.8	13.8	−4.80 (−11.13, 1.53)

BP, bodily pain; GH, general health; MH, mental health; PF, physical functioning; RE, role emotional; RP, role physical; SF, social functioning; VT, vitality

References are listed in Online Resource 5.

Supplemental Table 8. Overview of studies included in the meta-analysis of cytomegalovirus infection

		PEKT			Non-PEKT			
**Author**	**Year**	**No.**	**Total No.**	**%**	**No.**	**Total No.**	**%**	**Risk Ratio (95% CI)**
Okumi [16]	2017	18	93	19.4	16	93	17.2	1.13 (0.61–2.07)
Noda [23]	2016	1	7	14.3	6	16	37.5	0.38 (0.06–2.60)
Morales [25]	2015	3	26	11.5	4	26	15.4	0.75 (0.19–3.03)
Dębska-Ślizień [29]	2014	18	51	35.3	12	51	23.5	1.50 (0.81–2.78)
Oishi [28]	2015	4	25	16.0	12	61	19.7	0.81 (0.29–2.28)
Hayashida [36]	2013	20	29	69.0	11	15	73.3	0.94 (0.64–1.39)
Jung [43]	2010	24	62	38.7	148	390	37.9	1.02 (0.73–1.43)
Ishikawa [48]	2008	0	5	0.0	2	39	5.1	1.33 (0.07–24.54)
Ekstrand [73]	1993	2	24	8.3	1	101	1.0	8.42 (0.80–89.04)

References are listed in Online Resource 5.

Information Classification: General

Supplemental Table 9. Overview of studies included in the meta-analysis of urinary tract infection

		PEKT			Non-PEKT			
Author	Year	No.	Total No.	%	No.	Total No.	%	Risk Ratio (95% CI)
Morales [25]	2015	3	26	11.5	4	26	15.4	0.75 (0.19–3.03)
Dębska-Ślizień [29]	2014	24	51	47.1	25	51	49.0	0.96 (0.64–1.44)
Jung [43]	2010	2	62	3.2	34	390	8.7	0.37 (0.09–1.50)
Ishikawa [48]	2008	0	5	0.0	2	39	5.1	1.33 (0.07–24.54)

References are listed in Online Resource 5.

Information Classification: General

Supplemental Table 10. Subgroup analyses according to patient location, publication year, donor source, dialysis duration, and primary/repeated transplantation

		Patient mortality	Graft survival
Overall		HR 0.78 (0.66–0.92)<br>I^2 = 85%<br>10 studies	HR 0.81 (0.67–0.98)<br>I^2 = 93%<br>9 studies
Geographic Location	North America	HR 0.76 (0.63–0.93)<br>I^2 = 91%<br>5 studies	HR 0.91 (0.73–1.12)<br>I^2 = 95%<br>5 studies
	South America	N/A	N/A
	Oceania	HR 0.79 (0.52–1.21)<br>I^2 = 71%<br>3 studies	HR 0.71 (0.56–0.90)<br>1 study
	Asia	N/A	N/A
	Europe	HR 0.80 (0.32–1.98)<br>I^2 = 55%<br>2 studies	HR 0.63 (0.40–1.01)<br>I^2 = 73%<br>3 studies
	Africa	N/A	N/A
	Test for subgroup differences	Chi^2 = 0.03<br>*P* = 0.98<br>I^2 = 0%	Chi^2 = 3.26<br>*P* = 0.20<br>I^2 = 38.6%
Publication Year	2000 and before	N/A	N/A
	2001–2010	HR 0.79 (0.62–1.00)<br>I^2 = 82%<br>3 studies	HR 1.36 (1.20–1.54)<br>1 study
	2011 and later	HR 0.78 (0.59–1.02)<br>I^2 = 87%<br>7 studies	HR 0.75 (0.64–0.89)<br>I^2 = 89%<br>8 study
	Test for subgroup differences	Chi^2 = 0.01<br>*P* = 0.94<br>I^2 = 0%	Chi^2 = 32.24<br>*P* < 0.00001<br>I^2 = 96.9%
Donor Source	LD	HR 0.67 (0.50–0.91)<br>I^2 = 80%<br>4 studies	HR 0.75 (0.50–1.12)<br>I^2 = 94%<br>2 studies

Information Classification: General

	DD	HR 0.93 (0.87–0.99)<br>I^2 = 0%<br>3 studies	HR 0.81 (0.76–0.87)<br>I^2 = 0%<br>2 studies
	Mixed/unknown	HR 0.78 (0.57–1.07)<br>I^2 = 71%<br>4 studies	HR 0.78 (0.55–1.13)<br>I^2 = 96%<br>5 studies
	Test for subgroup differences	Chi^2 = 5.04<br>*P* = 0.08<br>I^2 = 60.3%	Chi^2 = 0.19<br>*P* = 0.91<br>I^2 = 0%
Dialysis Duration	<1.5 years	HR 0.94 (0.88–1.01)<br>I^2 = 0%<br>3 studies	HR 0.83 (0.74–0.93)<br>I^2 = 58%<br>3 studies
	Other	HR 0.70 (0.55–0.89)<br>I^2 = 86%<br>7 studies	HR 0.79 (0.56–1.10)<br>I^2 = 96%<br>6 studies
	Test for subgroup differences	Chi^2 = 5.20<br>*P* = 0.02<br>I^2 = 80.8%	Chi^2 = 0.07<br>*P* = 0.79<br>I^2 = 0%
Repeated Transplantation	Primary	HR 0.76 (0.62–0.92)<br>I^2 = 85%<br>8 studies	HR 0.73 (0.62–0.87)<br>I^2 = 87%<br>6 studies
	Repeated	HR 0.81 (0.41–1.62)<br>I^2 = 57%<br>2 studies	HR 1.00 (0.72–1.41)<br>I^2 = 91%<br>3 studies
	Test for subgroup differences	Chi^2 = 0.04<br>*P* = 0.85<br>I^2 = 0%	Chi^2 = 2.60<br>*P* = 0.11<br>I^2 = 61.5%

HR, hazard ratio; N/A, not available

Information Classification: General

Supplemental Table 11. Sensitivity analyses using the leave-one-out method

Patient mortality		Graft survival		Cardiovascular disease		Acute rejection		Cytomegalovirus infection		Urinary tract infection	
**(Overall)**	HR 0.78 (0.66–0.92) I^2 = 85% 10 studies	**(Overall)**	HR 0.81 (0.67–0.98) I^2 = 93% 9 studies	**(Overall)**	RR 0.90 (0.58–1.40) I^2 = 0% 6 studies	**(Overall)**	RR 0.75 (0.55–1.03) I^2 = 36% 9 studies	**(Overall)**	RR 1.04 (0.85–1.29) I^2 = 0% 9 studies	**(Overall)**	RR 0.89 (0.61–1.29) I^2 = 0% 4 studies
**Omitted Study**		**Omitted Study**		**Omitted Study**		**Omitted Study**		**Omitted Study**		**Omitted Study**	
Irish [5] 2019	HR 0.75 (0.63–0.90) I^2 = 86%	Foucher [7] 2019	HR 0.79 (0.65–0.97) I^2 = 94%	Okumi [16] 2017	RR 0.94 (0.60–1.50) I^2 = 0%	Girerd [14] 2018	RR 0.82 (0.62–1.09) I^2 = 19%	Okumi [16] 2017	RR 1.03 (0.83–1.29) I^2 = 0%	Morales [25] 2015	RR 0.90 (0.61–1.32) I^2 = 0%
Girerd [14] 2018	HR 0.79 (0.67–0.93) I^2 = 86%	Prezelin-Reydit [8] 2019	HR 0.86 (0.71–1.03) I^2 = 92%	Goto [21] 2016	RR 0.92 (0.58–1.44) I^2 = 0%	Morales [25] 2015	RR 0.74 (0.56–0.96) I^2 = 20%	Noda [23] 2016	RR 1.06 (0.85–1.30) I^2 = 0%	Debska-Slizien [29] 2015	RR 0.58 (0.23–1.48) I^2 = 0%
Haller [15] 2017	HR 0.77 (0.65–0.92) I^2 = 86%	Gill [12] 2018	HR 0.79 (0.63–0.99) I^2 = 94%	Hayashida [36] 2013	RR 0.93 (0.60–1.45) I^2 = 0%	Kohei [30] 2014	RR 0.72 (0.50–1.04) I^2 = 41%	Morales [25] 2015	RR 1.05 (0.85–1.30) I^2 = 0%	Jung [43] 2010	RR 0.95 (0.65–1.39) I^2 = 0%
Jay [22] 2016	HR 0.84 (0.74–0.96) I^2 = 67%	Girerd [14] 2018	HR 0.83 (0.69–1.01) I^2 = 94%	Son [42] 2010	RR 0.96 (0.61–1.52) I^2 = 0%	Sayin [32] 2013	RR 0.76 (0.52–1.10) I^2 = 44%	Oishi [28] 2015	RR 1.05 (0.85–1.31) I^2 = 0%	Ishikawa [48] 2008	RR 0.88 (0.61–1.28) I^2 = 0%

Information Classification: General

Grams [35] 2013	HR 0.75 (0.61–0.92) I^2 = 83%	Haller [15] 2017	HR 0.82 (0.67–1.01) I^2 = 94%	Innocenti [52] 2007	RR 0.80 (0.45–1.43) I^2 = 0%	Luo [37] 2012	RR 0.80 (0.59–1.08) I^2 = 31%	Debska-Slizien [29] 2015	RR 1.00 (0.80–1.24) I^2 = 0%
Naveed [39] 2011	HR 0.80 (0.68–0.95) I^2 = 85%	Jay [22] 2016	HR 0.84 (0.69–1.03) I^2 = 93%	Ekstrand [73] 1993	RR 0.81 (0.48–1.37) I^2 = 0%	Son [42] 2010	RR 0.75 (0.54–1.05) I^2 = 44%	Hayashida [36] 2013	RR 1.09 (0.85–1.40) I^2 = 0%
Kessler [41] 2011	HR 0.77 (0.65–0.91) I^2 = 86%	Johnston [34] 2013	HR 0.78 (0.63–0.98) I^2 = 94%			Jung [43] 2010	RR 0.69 (0.50–0.95) I^2 = 25%	Jung [43] 2010	RR 1.06 (0.81–1.38) I^2 = 0%
Milton [47] 2008	HR 0.80 (0.68–0.95) I^2 = 85%	Grams [35] 2013	HR 0.70 (0.63–1.02) I^2 = 94%			Ishikawa [48] 2008	RR 0.75 (0.54–1.04) I^2 = 43%	Ishikawa [48] 2008	RR 1.04 (0.84–1.29) I^2 = 0%
Goldfarb [55] 2006	HR 0.75 (0.62–0.90) I^2 = 84%	Goldfarb [55] 2006	HR 0.75 (0.64–0.89) I^2 = 89%			Innocenti [52] 2007	RR 0.77 (0.53–1.12) I^2 = 42%	Ekstrand [73] 1993	RR 1.03 (0.83–1.27) I^2 = 0%
Kasiske [65] 2002 [LD]	HR 0.79 (0.66–0.95) I^2 = 85%								
Kasiske [65] 2002 [DD]	HR 0.77 (0.64–0.93)								

Information Classification: General

I^2 = 86%					

DD, deceased donor transplantation; HR, hazard ratio; LD, living donor transplantation; RR, relative risk

References are listed in Online Resource 5.

Information Classification: General

Supplemental Table 12. Risk of bias assessment in individual studies

Author, Year	Outcome	Pre-intervention		At intervention	Post-intervention				Overall
		Bias due to confounding	Bias in selection of participants in to study	Bias in classification of interventions	Bias due to deviations from intended interventions	Bias due to missing data	Bias in measurement of outcomes	Bias in selection of the reported result	
Auneau-Enjalbert [1], 2021	QOL	Serious	Low	Low	Low	Moderate	Low	Low	Serious
Aytekin [2], 2020	Patient survival	Serious	Low	Low	Low	No Information	Low	Low	Serious
	Acute rejection	Serious				No Information	Low	Low	Serious
Mitsui [3], 2020	QOL	Serious	Low	Low	Low	Serious	Low	Low	Serious
Franco [4], 2020	Patient survival	Serious	Low	Low	Low	No Information	Low	Low	Serious
	Graft survival	Serious				No Information	Low	Low	Serious
	Acute rejection	Serious				No Information	Low	Low	Serious
Irish [5], 2019	Patient survival	Moderate	Low	Low	Low	Low	Low	Low	Moderate
Kim [6], 2019	Patient survival	Moderate	Low	Low	Low	No Information	Low	Low	Moderate
	Graft survival	Moderate				No Information	Low	Low	Moderate
Foucher [7], 2019	Graft survival	Moderate	Low	Low	Low	No Information	Low	Low	Moderate

Information Classification: General

Prezelin-Reydit [8], 2019	Patient survival	Serious	Low	Low	Low	No Information	Low	Low	Serious
	Graft survival	Serious				No Information	Low	Low	Serious
Mochizuki [9], 2019	Graft survival	Moderate	Low	Low	Low	No Information	Low	Moderate	Moderate
	Acute rejection	Moderate				No Information	No Information	No Information	No Information
Matsumura [10], 2018	QOL	Serious	Low	Low	Low	Low	Low	Low	Serious
Aufhauser [11], 2018	Patient survival	Critical	Low	Low	Low	No Information	Low	Low	Critical
	Graft survival	Critical				No Information	Low	Low	Critical
Gill [12], 2018	Patient survival	Moderate	Low	Low	Low	No Information	Low	Low	Moderate
	Graft survival	Moderate				No Information	Low	Low	Moderate
Mursawa [13], 2018	Graft survival	Critical	Low	Low	Low	No Information	Low	Low	Critical
Girerd [14], 2018	Patient survival	Moderate	Low	Low	Low	No Information	Low	Low	Moderate
	Graft survival	Moderate				No Information	Low	Low	Moderate
	Acute rejection	Serious				No Information	Low	Low	Serious
Haller [15], 2017	Patient survival	Serious	Low	Low	Moderate	Low	Low	Low	Serious
	Graft survival	Serious				Low	Low	Low	Serious

Information Classification: General

Okumi [16], 2017	Patient survival	Moderate	Low	Low	Low	No Information	Low	No Information	No Information
	Graft survival	Moderate				No Information	Low	Low	Moderate
	CVD	Moderate				No Information	Moderate	Low	Moderate
	Infection	Moderate				No Information	Low	Low	Moderate
Nakagawa [17], 2017	Patient survival	Serious	Low	Low	Low	No Information	Low	No Information	No Information
	Graft survival	Serious				No Information	Low	No Information	No Information
Gadelkareem [18], 2017	Patient survival	Serious	Low	Low	Low	Moderate	Low	No Information	Serious
	Graft survival	Serious				Moderate	Low	No Information	Serious
	Acute rejection	Serious				Moderate	Low	No Information	Serious
Girerd [19], 2016	Graft survival	Serious	Low	Low	Low	No Information	Low	Low	Serious
Bzoma [20], 2016	QOL	Serious	Low	Low	Low	No Information	Serious	Low	Serious
Goto [21], 2016	Patient survival	Serious	Low	Low	Low	No Information	Low	Low	Serious
	Graft survival	Serious				No Information	Low	Low	Serious
	CVD	Critical				No Information	Low	Low	Critical
Jay [22], 2016	Patient survival	Moderate	Low	Low	Low	No Information	Low	Low	Moderate

Information Classification: General

	Graft survival	Moderate				No Information	Low	Low	Moderate
Noda [23], 2016	Acute rejection	Serious	Low	Low	Moderate	No Information	Moderate	No Information	No Information
	Infection	Serious				No Information	Moderate	No Information	No Information
Florit [24], 2015	Patient survival	Critical	Low	Low	Low	No Information	Low	Low	Critical
	Graft survival	Critical				No Information	Low	Low	Critical
	Acute rejection	Critical				No Information	Low	Low	Critical
Morales [25], 2015	Patient survival	Serious	Low	Low	Low	Low	Low	Low	Serious
	Graft survival	Serious				Low	Low	Low	Serious
	Acute rejection	Serious				Low	Low	Low	Serious
	Infection	Serious				Low	Low	Low	Serious
Unsal [26], 2015	Patient survival	Serious	Low	Low	Low	No Information	Low	Low	Serious
	Acute rejection	Serious				No Information	Low	Low	Serious
Nakamura [27], 2015	Patient survival	Critical	Low	Low	Low	No Information	Low	No Information	No Information
	Graft survival	Critical				No Information	Low	No Information	No Information
	Acute rejection	Critical				No Information	Low	No Information	No Information

	Infection	Critical				No Information	Low	No Information	No Information
Oishi [28], 2015	Patient survival	Serious	Low	Low	Low	No Information	Low	No Information	No Information
	Acute rejection	Serious				No Information	Moderate	No Information	No Information
	Infection	Serious				No Information	Moderate	No Information	No Information
Dębska-Ślizień [29], 2014	Patient survival	Serious	Low	Low	Low	No Information	Low	No Information	No Information
	Graft survival	Serious				No Information	Low	No Information	No Information
	Acute rejection	Serious				No Information	Low	No Information	No Information
	Infection	Serious				No Information	Low	No Information	No Information
Kohei [30], 2014	Patient survival	Critical	Low	Low	Low	No Information	Low	Low	Critical
	Graft survival	Serious				No Information	Low	Low	Serious
	Acute rejection	Serious				No Information	Low	Low	Serious
Ryosaka [31], 2014	Patient survival	Serious	Low	Low	Low	No Information	Low	Low	Serious
	Graft survival	Serious				No Information	Low	Low	Serious
Sayin [32], 2013	Patient survival	Serious	Low	Low	Low	No Information	Low	Low	Serious
	Graft survival	Serious				No Information	Low	Low	Serious

Information Classification: General

	Acute rejection	Serious				No Information	Low	Low	Serious
	Infection	Serious				No Information	Low	Low	Serious
Bozkurt [33], 2013	Patient survival	Serious	Low	Low	Low	No Information	Low	Low	Serious
	Graft survival	Serious				No Information	Low	Low	Serious
Johnston [34], 2013	Patient survival	Moderate	Low	Low	Low	No Information	Low	Low	Moderate
	Graft survival	Moderate				No Information	Low	Low	Moderate
	Acute rejection	Critical				No Information	Low	Low	Critical
Grams [35], 2013	Patient survival	Moderate	Low	Low	Low	No Information	Low	Low	Moderate
	Graft survival	Moderate				No Information	Low	Low	Moderate
Hayashida [36], 2013	Graft survival	Moderate	Low	Low	Low	No Information	Low	No Information	No Information
	CVD	Moderate				No Information	Moderate	No Information	No Information
	Acute rejection	Moderate				No Information	Moderate	No Information	No Information
	Infection	Moderate				No Information	Moderate	No Information	No Information
Luo [37], 2012	Patient survival	Moderate	Low	Low	Low	No Information	Low	Low	Moderate
	Graft survival	Moderate				No Information	Low	Low	Moderate

	Acute rejection	Moderate				No Information	Low	Low	Moderate
Keith [38], 2012	Patient survival	Serious	Low	Low	Low	No Information	Low	Low	Serious
	Graft survival	Serious				No Information	Low	Low	Serious
Naveed [39], 2011	Patient survival	Serious	Low	Low	Low	No Information	Low	Low	Serious
	Graft survival	Serious				No Information	Low	Low	Serious
Rigo [40], 2011	Acute rejection	Serious	Low	Low	Low	No Information	Low	Low	Serious
Kessler [41], 2011	Patient survival	Moderate	Low	Low	Low	No Information	Low	Low	Moderate
	Graft survival	Moderate				No Information	Low	Low	Moderate
	Acute rejection	Serious				No Information	Low	Low	Serious
Son [42], 2010	Patient survival	Serious	Low	Low	Low	No Information	Low	Low	Serious
	Graft survival	Serious				No Information	Low	Low	Serious
	CVD	Serious				No Information	Low	No Information	No Information
	Acute rejection	Serious				No Information	Low	No Information	No Information
	Infection	Serious				No Information	Low	No Information	No Information
Jung [43], 2010	Patient survival	Serious	Low	Low	Low	No Information	Low	No Information	No Information

	Graft survival	Serious				No Information	Low	Low	Serious
	Acute rejection	Critical				No Information	Low	Low	Critical
	infection	Serious				No Information	Low	Low	Serious
Witczak [44], 2009	Patient survival	Moderate	Low	Low	Low	Low	Low	Low	Moderate
	Graft survival	Moderate				Low	Low	Low	Moderate
Salvadori [45], 2009	Patient survival	Serious	Low	Low	Low	No Information	Low	Low	Serious
	Graft survival	Serious				No Information	Low	Low	Serious
Yoo [46], 2009	Patient survival	Serious	Low	Low	Low	No Information	Low	Low	Serious
	Graft survival	Serious				No Information	Low	Low	Serious
	Acute rejection	Serious				No Information	Low	Low	Serious
Milton [47], 2008	Patient survival	Moderate	Low	Low	Low	No Information	Low	Low	Moderate
	Graft survival	Moderate				No Information	Low	Low	Moderate
	Acute rejection	Serious				No Information	Low	Low	Serious
Ishikawa [48], 2008	Patient survival	Serious	Low	Low	Low	No Information	Low	No Information	No Information
	Graft survival	Serious				No Information	Low	No Information	No Information

Information Classification: General

	Acute rejection	Serious				No Information	Low	No Information	No Information
	Infection	Serious				No Information	Low	No Information	No Information
Joo [49], 2007	Patient survival	Serious	Low	Low	Low	Low	Low	Low	Serious
	Graft survival	Serious				Low	Low	Low	Serious
	Acute rejection	Serious				Low	Low	Low	Serious
	Infection	Serious				Low	Moderate	No Information	Serious
Pour-Reza-Gholi [50], 2007	Patient survival	Serious	Low	Low	Low	No Information	Low	Low	Serious
	Graft survival	Serious				No Information	Low	Low	Serious
	Acute rejection	Serious				No Information	Low	Low	Serious
Pérez-Flores [51], 2007	Patient survival	Serious	Low	Low	Low	No Information	Low	low	Serious
	Graft survival	Serious				No Information	Low	Low	Serious
	Acute rejection	Serious				No Information	Low	No Information	No Information
Innocenti [52], 2007	Patient survival	Serious	Low	Low	Low	No Information	Low	Low	Serious
	Graft survival	Serious				No Information	Low	Low	Serious
	CVD	Serious				No Information	Low	Low	Serious

Information Classification: General

	Acute rejection	Serious				No Information	Low	Low	Serious
	Infection	Serious				No Information	Moderate	Low	Moderate
Kennedy [53], 2006	Graft survival	Serious	Low	Low	Low	No Information	Low	Low	Serious
Dębska-Ślizień [54], 2006	Patient survival	Serious	Low	Low	Low	No Information	Low	No Information	No Information
	Graft survival	Serious				No Information	Low	No Information	No Information
	Acute rejection	Serious				No Information	Low	No Information	No Information
Goldfarb-Rumyantzev [55], 2006	Patient survival	Moderate	Low	Low	Low	No Information	Low	Low	Moderate
	Graft survival	Moderate				No Information	Low	Low	Moderate
Becker [56], 2006	Patient survival	Serious	Low	Low	Low	Low	Low	Low	Serious
	Graft survival	Serious				Low	Low	Low	Serious
Abou Ayache [57], 2005	Patient survival	Critical	Low	Low	Low	No Information	Low	No Information	No Information
	Graft survival	Critical				No Information	Low	No Information	No Information
Goldfarb-Rumyantzev [58], 2005	Patient survival	Moderate	Low	Low	Low	No Information	Low	Low	Moderate
	Graft survival	Moderate				No Information	Low	Low	Moderate

Gill [59], 2004	Patient survival	Serious	Low	Low	Low	No Information	Low	Low	Serious
	Graft survival	Serious				No Information	Low	Low	Serious
	Acute rejection	Serious				No Information	Low	No Information	No Information
el-Agroudy [60], 2004	Patient survival	Serious	Low	Low	Low	Low	Low	Low	Serious
	Graft survival	Serious				Low	Low	Low	Serious
	Acute rejection	Serious				Low	Low	Low	Serious
	Infection	Serious				Low	Moderate	No Information	Serious
Simforoosh [61], 2003	Patient survival	Serious	Low	Low	Low	No Information	Low	Low	Serious
	Graft survival	Serious				No Information	Low	Low	Serious
	Acute rejection	Serious				No Information	Low	Low	Serious
Mange [62], 2003	Acute rejection	Serious	Low	Low	Low	Low	Low	Low	Serious
Nishikawa [63], 2002	Patient survival	Critical	Low	Low	Low	No Information	Low	Low	Critical
	Graft survival	Critical				No Information	Low	Low	Critical
	Acute rejection	Critical				No Information	Low	Low	Critical
Meier-Kriesche [64], 2002	Graft survival	Serious	Low	Low	Low	No Information	Low	Low	Serious

Information Classification: General

Kasiske [65], 2002	Patient survival	Serious	Low	Low	Low	No Information	Low	Low	Serious
	Graft survival	Serious				No Information	Low	Low	Serious
Mange [66], 2001	Patient survival	Serious	Low	Low	Low	No Information	Low	Low	Serious
	Graft survival	Serious				No Information	Low	Low	Serious
Meier-Kriesche [67], 2000	Patient survival	Moderate	Low	Low	Low	No Information	Low	Low	Moderate
	Graft survival	Moderate				No Information	Low	Low	Moderate
Papalois [68], 2000	Patient survival	Serious	Low	Low	Low	No Information	Low	Low	Serious
	Graft survival	Serious				No Information	Low	Low	Serious
	Acute rejection	Serious				No Information	Low	Low	Serious
	QOL	Serious				No Information	Low	Low	Serious
John [69], 1998	Patient survival	Serious	Low	Low	Low	No Information	Low	Low	Serious
	Graft survival	Serious				No Information	Low	Low	Serious
Asderakis [70], 1998	Patient survival	Serious	Low	Low	Low	No Information	Low	Low	Serious
	Graft survival	Serious				No Information	Low	Low	Serious
	Acute rejection	Serious				No Information	Low	Low	Serious

Information Classification: General

Roake [71], 1996	Patient survival	Moderate	Low	Low	Low	No Information	Low	Low	Moderate
	Graft survival	Moderate				No Information	Low	Low	Moderate
Berthoux [72], 1996	Patient survival	No Information	Low	Low	Low	No Information	Low	Low	No Information
	Graft survival	No Information				No Information	Low	Low	No Information
Ekstrand [73], 1993	Patient survival	Serious	Low	Low	Low	No Information	Low	Low	Serious
	Graft survival	Serious				No Information	Low	Low	Serious
	CVD	Serious				No Information	Low	Low	Serious
	Acute rejection	Serious				No Information	Low	Low	Serious
	infection	Serious				No Information	Moderate	Low	Serious
Cacciarelli [74], 1993	Patient survival	Critical	Low	Low	Low	No Information	Low	Low	Critical
	Graft survival	Critical				No Information	Low	Low	Critical
	Acute rejection	Critical				No Information	Low	Low	Critical
Katz [75], 1991	Patient survival	Serious	Moderate	Low	Low	No Information	Low	Low	Serious
	Graft survival	Serious				No Information	Low	Low	Serious
	Acute rejection	Serious				No Information	Low	Low	Serious

Migliori [76], 1987	Patient survival	No Information	Low	Low	Low	No Information	Low	Low	No Information
	Graft survival	No Information				No Information	Low	Low	No Information

CVD, cardiovascular disease; QOL, quality of life

References are listed in Supplemental Item 5.

Information Classification: General
