# Supplementary material for: Efficacy of pre-emptive kidney transplantation for adults with end-stage kidney disease: a systematic review and meta-analysis
Source: Ren Fail. 2023 Jan 27;45(1):2169618. doi: 10.1080/0886022X.2023.2169618 (PMC9888453; doi:10.1080/0886022X.2023.2169618)
Supplement: Supplemental Material [file IRNF_A_2169618_SM2272.pdf]

Supplemental Fig 1. Forest plot of all-cause mortality in the donor type subgroup

Study	HR [95% CI]	Weight
**Living Donor**		
Irish 2019	1.12 [0.78, 1.61]	8.4%
Jay 2016	0.55 [0.47, 0.64]	12.6%
Milton 2008	0.46 [0.26, 0.80]	5.5%
Kasiske 2002 [LD]	0.69 [0.56, 0.85]	11.5%
**Subtotal**	**0.67 [0.50, 0.91]**	**37.9%**
Heterogeneity; I^2 = 80%		
**Deceased Donor**		
Grams 2013	0.94 [0.87, 1.01]	13.7%
Kessler 2011	1.20 [0.57, 2.51]	3.7%
Kasiske 2002 [DD]	0.84 [0.71, 0.99]	12.3%
**Subtotal**	**0.93 [0.87, 0.99]**	**29.7%**
Heterogeneity; I^2 = 0%		
**Mixed / Unknown**		
Girerd 2018	0.47 [0.18, 1.26]	2.3%
Haller 2017	0.84 [0.62, 1.13]	9.7%
Naveed 2011	0.55 [0.36, 0.84]	7.3%
Goldfarb 2006	1.02 [0.90, 1.15]	13.1%
**Subtotal**	**0.78 [0.57, 1.07]**	**32.4%**
Heterogeneity; I^2 = 71%		
**Total**	**0.78 [0.66, 0.92]**	**100.0%**
Heterogeneity; I^2 = 85%		

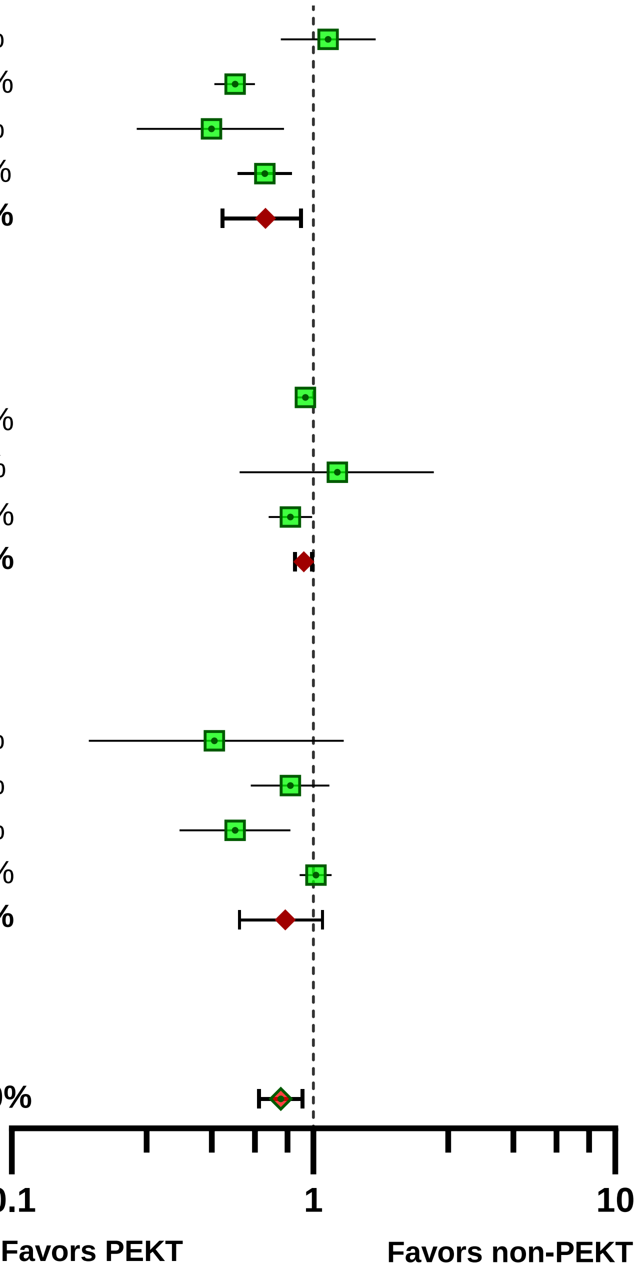

Information Classification: General

Supplemental Fig 2. Forest plot of death with functioning graft

Study	HR [95% CI]	Weight
Prezelin 2019	0.60 [0.51, 0.71]	31.3%
Gill 2018	0.86 [0.75, 0.98]	34.5%
Johnston 2013	0.76 [0.66, 0.87]	34.1%
**Total**	**0.74 [0.61, 0.89]**	**100.0%**

Heterogeneity; $I^2 = 82\%$

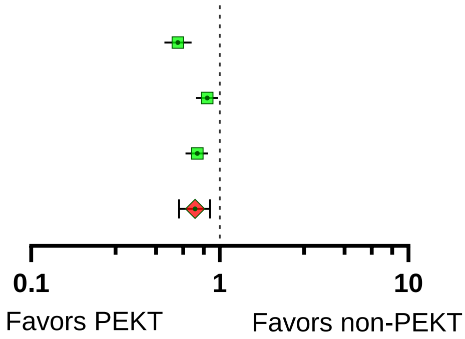

Information Classification: General

Supplemental Fig 3. Forest plot of cardiovascular disease

Study	RR [95% CI]	Weight
Okumi 2017	0.60 [0.15, 2.44]	9.8%
Goto 2016	0.76 [0.16, 3.75]	7.6%
Hayashida 2013	0.18 [0.01, 4.12]	1.9%
Son 2010	0.44 [0.10, 2.05]	8.2%
Innocenti 2007	1.06 [0.54, 2.11]	41.3%
Ekstrand	1.15 [0.52, 2.52]	31.1%
**Total**	**0.90 [0.58, 1.40]**	**100.0%**

Heterogeneity; I^2 = 0%

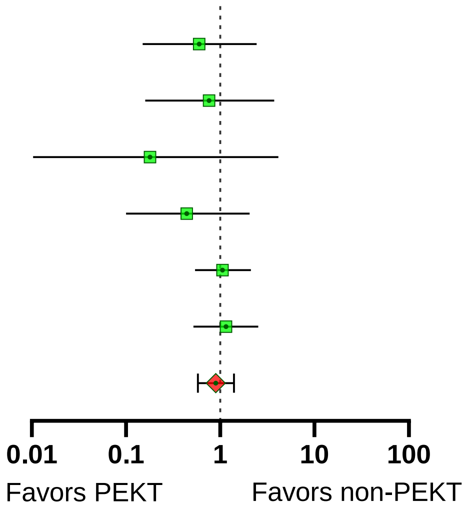

Information Classification: General

Supplemental Fig 4. Forest plot of biopsy-proven acute rejection

Study	RR [95% CI]	Weight
Girerd 2018	0.40 [0.19, 0.83]	12.0%
Morales 2015	6.00 [0.78, 46.42]	2.1%
Kohei 2014	0.92 [0.53, 1.61]	16.4%
Sayin 2013	0.73 [0.42, 1.25]	16.8%
Luo 2012	0.38 [0.15, 0.99]	8.1%
Son 2010	0.80 [0.21, 3.09]	4.5%
Jung 2010	1.09 [0.69, 1.73]	19.6%
Ishikawa 2008	0.97 [0.15, 6.26]	2.6%
Innocenti 2007	0.66 [0.40, 1.10]	18.0%
**Total**	**0.75 [0.55, 1.03]**	**100.0%**

Heterogeneity; I^2 = 36%

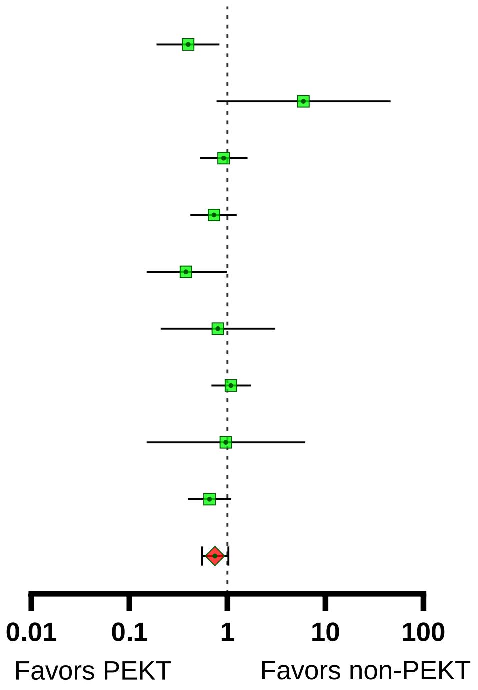

Information Classification: General

Supplemental Fig 5. Forest plot of quality of life (SF-36)

Study	MD [95% CI]	Weight
Auneau-Enjalbert 2021	1.44 [−3.70, 6.58]	42.0%
Mitsui 2020	−1.20 [−7.65, 5.25]	26.7%
Matsumura 2018	−0.70 [−6.65, 5.25]	31.3%
**Total (PF)**	**0.06 [−3.27, 3.39]**	**100.0%**
Heterogeneity; I^2 = 0%		
Auneau-Enjalbert 2021	5.32 [−3.79, 14.43]	28.5%
Mitsui 2020	−2.50 [−8.60, 3.60]	49.7%
Matsumura 2018	−5.20 [−15.96, 5.56]	21.8%
**Total (RP)**	**−0.86 [−6.38, 4.66]**	**100.0%**
Heterogeneity; I^2 = 25%		
Auneau-Enjalbert 2021	3.36 [−1.89, 8.61]	37.7%
Mitsui 2020	−7.00 [−13.39, −0.61]	33.6%
Matsumura 2018	−0.60 [−8.49, 7.29]	28.7%
**Total (BP)**	**−1.26 [−7.72, 5.19]**	**100.0%**
Heterogeneity; I^2 = 67%		
Auneau-Enjalbert 2021	1.41 [−2.71, 5.53]	52.3%
Mitsui 2020	−1.60 [−7.12, 3.92]	29.1%
Matsumura 2018	−2.20 [−9.10, 4.70]	18.6%
**Total (GH)**	**−0.14 [−3.12, 2.84]**	**100.0%**
Heterogeneity; I^2 = 0%		
Auneau-Enjalbert 2021	1.36 [−2.93, 5.65]	56.3%
Mitsui 2020	0.20 [−5.66, 6.06]	30.1%
Matsumura 2018	−3.30 [−12.03, 5.43]	13.6%
**Total (VT)**	**0.38 [−2.84, 3.60]**	**100.0%**
Heterogeneity; I^2 = 0%		
Auneau-Enjalbert 2021	−0.75 [−5.62, 4.12]	43.6%
Mitsui 2020	−2.30 [−7.48, 2.88]	38.6%
Matsumura 2018	−4.90 [−12.54, 2.74]	17.7%
**Total (SF)**	**−2.08 [−5.30, 1.13]**	**100.0%**
Heterogeneity; I^2 = 0%		
Auneau-Enjalbert 2021	5.41 [−3.56, 14.38]	31.2%
Mitsui 2020	−2.30 [−8.03, 3.43]	50.5%
Matsumura 2018	−7.60 [−20.60, 5.40]	18.3%
**Total (RE)**	**−0.86 [−7.10, 5.38]**	**100.0%**
Heterogeneity; I^2 = 36%		
Auneau-Enjalbert 2021	0.88 [−2.86, 4.62]	55.3%
Mitsui 2020	−1.20 [−6.72, 4.32]	25.4%
Matsumura	−4.80 [−11.13, 1.53]	19.3%
**Total (MH)**	**−0.75 [−3.53, 2.04]**	**100.0%**
Heterogeneity; I^2 = 14%		

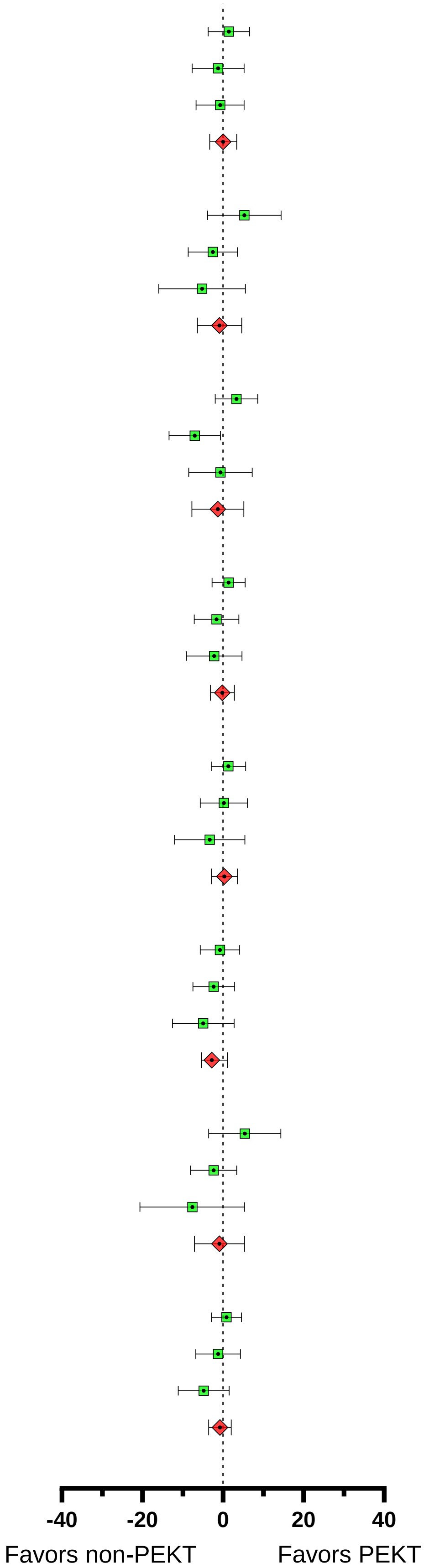

Information Classification: General

Supplemental Fig 6. Forest plot of cytomegalovirus infection

Study	RR [95% CI]	Weight
Okumi2017	1.13 [0.61, 2.07]	11.9%
Noda 2016	0.38 [0.06, 2.60]	1.2%
Morales 2015	0.75 [0.19, 3.03]	2.3%
Debska 2015	1.50 [0.81, 2.78]	11.5%
Oishi 2015	0.81 [0.29, 2.28]	4.2%
Hayashida 2013	0.94 [0.64, 1.39]	28.9%
Jung 2010	1.02 [0.73, 1.43]	38.7%
Ishikawa 2008	1.33 [0.07, 24.54]	0.5%
Ekstrand 1993	8.42 [0.80, 89.04]	0.8%
**Total**	**1.04 [0.85, 1.29]**	**100.0%**

Heterogeneity; $I^2 = 0\%$

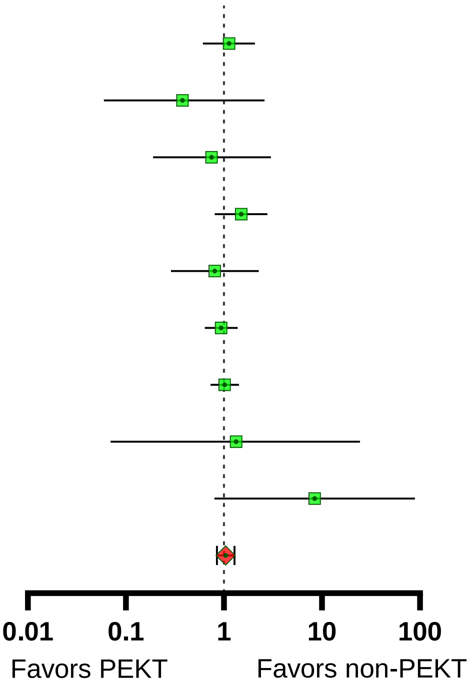

Information Classification: General

Supplemental Fig 7. Forest plot of urinary tract infection

Study	RR [95% CI]	Weight
Morales 2015	0.75 [0.19, 3.03]	7.1%
Debska 2015	0.96 [0.64, 1.44]	84.3%
Jung 2010	0.37 [0.09, 1.50]	7.0%
Ishikawa 2008	1.33 [0.07, 24.54]	1.6%
**Total**	**0.89 [0.61, 1.29]**	**100.0%**

Heterogeneity; $I^2 = 0\%$

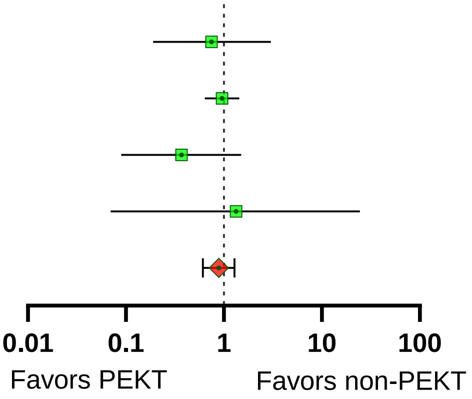

Information Classification: General

Supplemental Fig 8. Risk of bias assessment of patient mortality

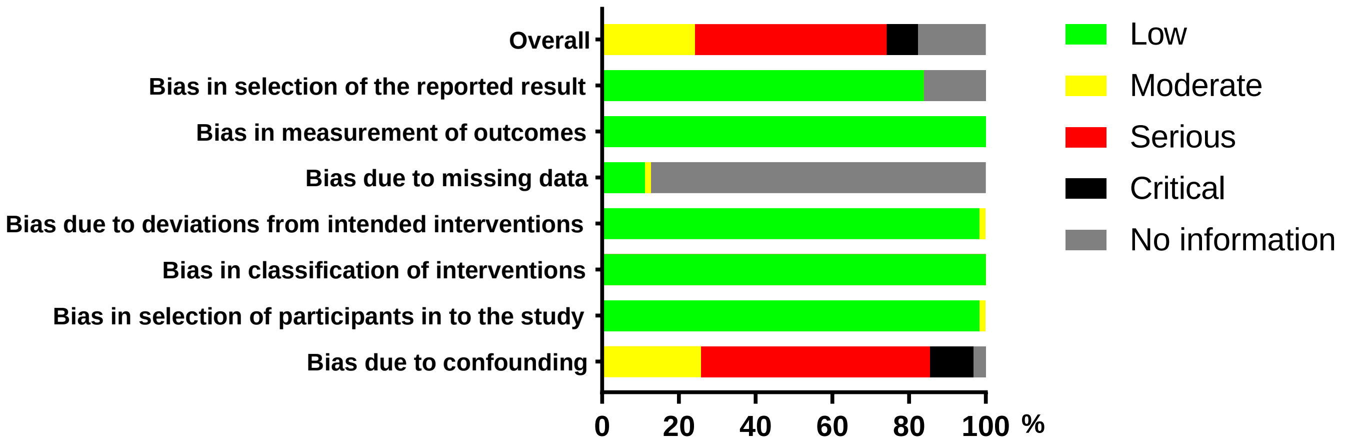

Information Classification: General

Supplemental Fig 9. Risk of bias assessment of graft survival

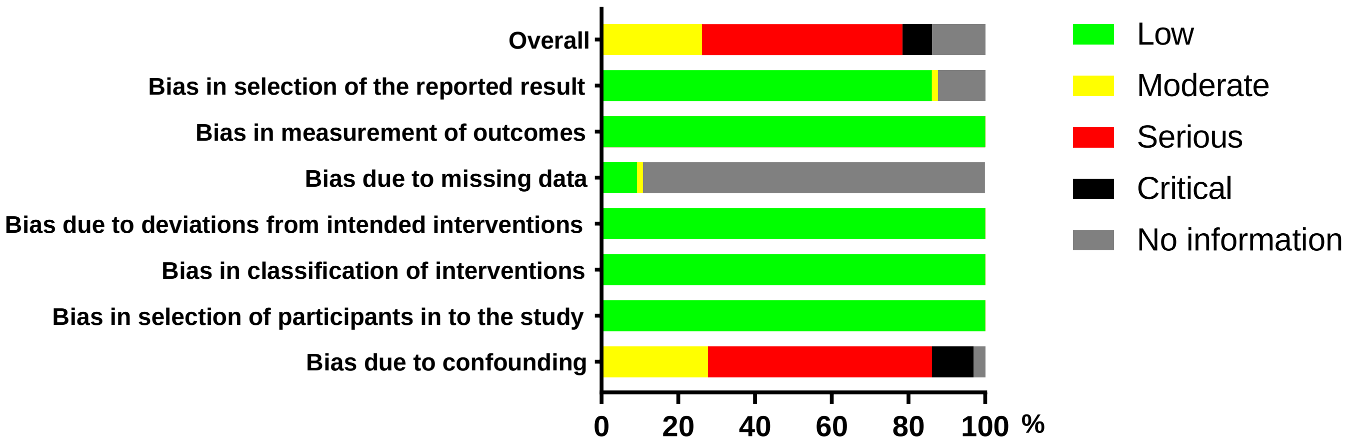

Information Classification: General

Supplemental Fig 10. Risk of bias assessment of cardiovascular disease

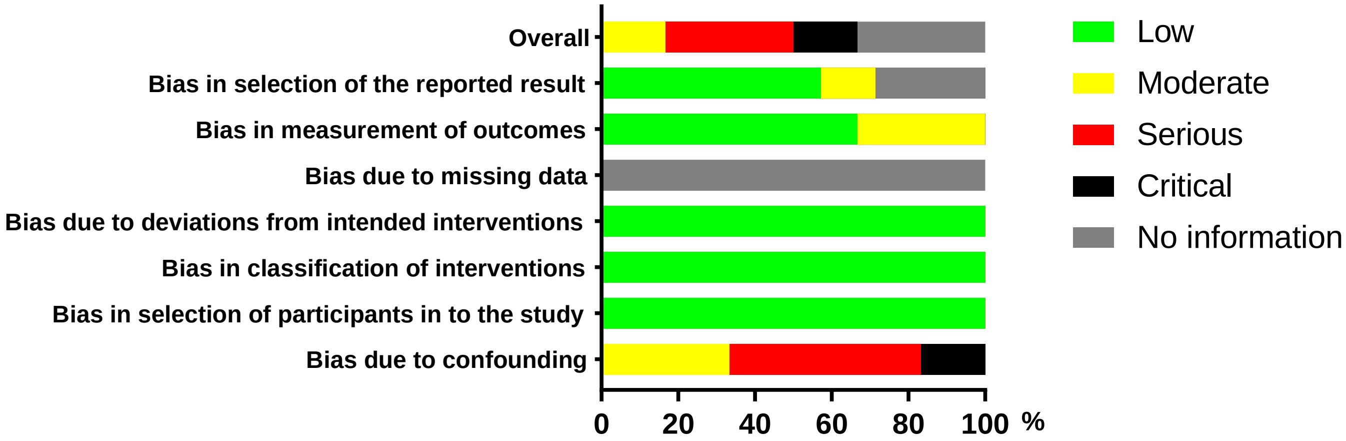

Supplemental Fig 11. Risk of bias assessment of biopsy-proven acute rejection

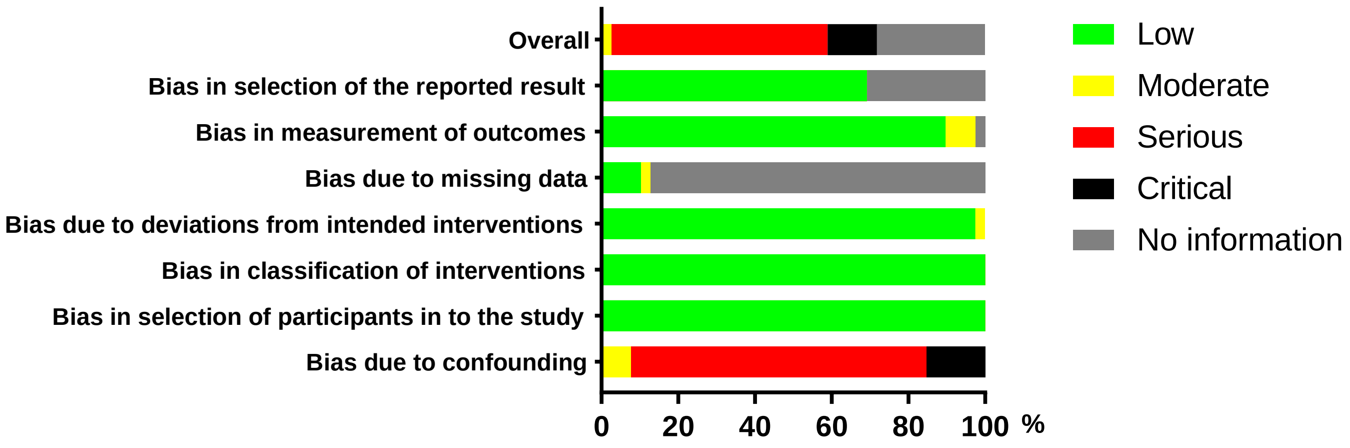

Supplemental Fig 12. Risk of bias assessment of infections

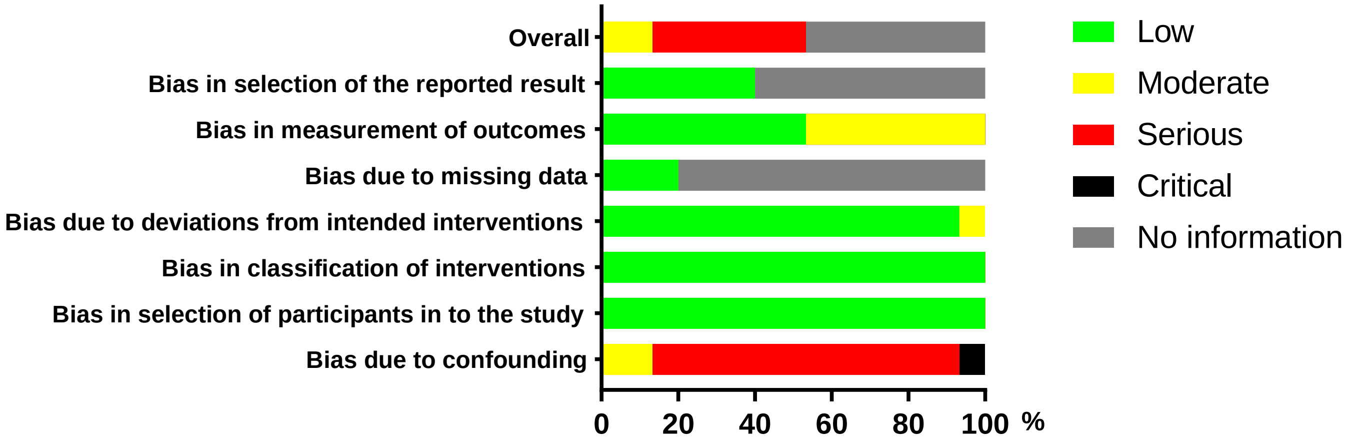

Information Classification: General

Supplemental Fig 13. Risk of bias assessment of quality of life

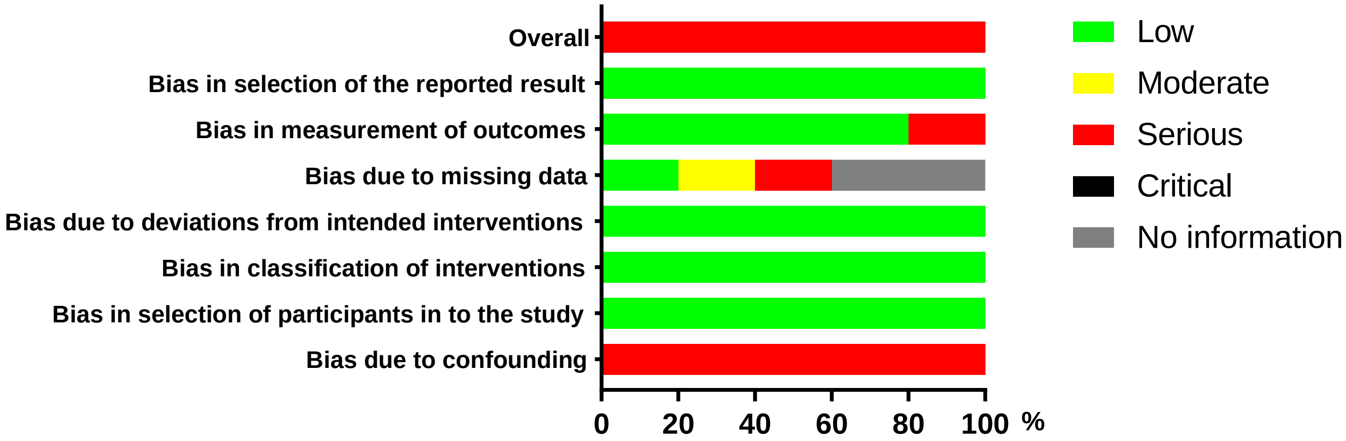

Information Classification: General

Supplemental Fig 14. Funnel plot of patient mortality

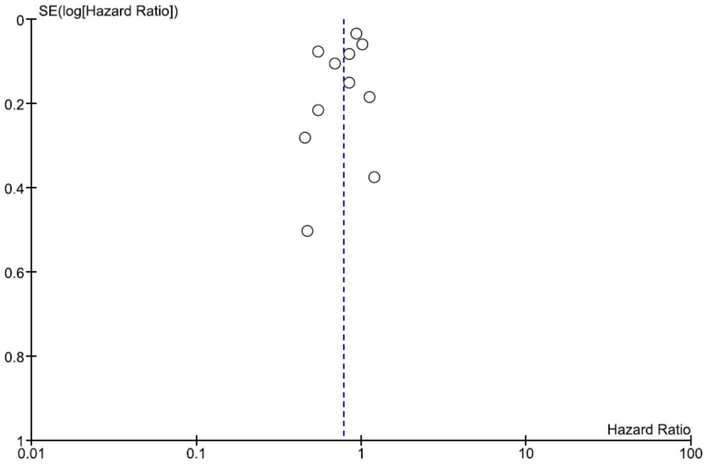

Supplemental Fig 15. Funnel plot of graft survival

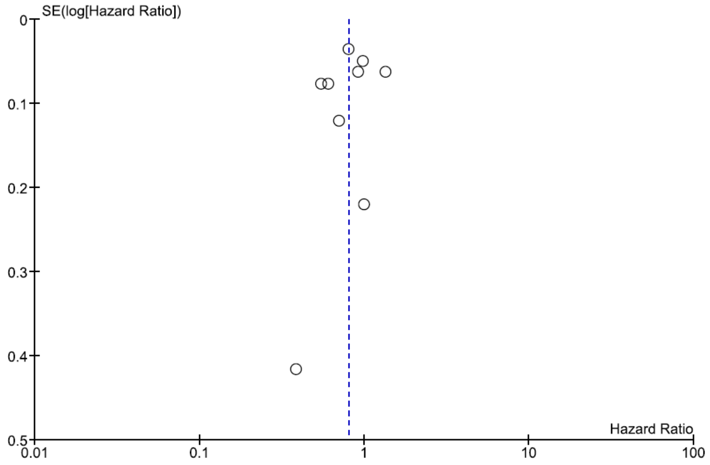

Information Classification: General

Supplemental Fig 16. Funnel plot of cardiovascular diseases

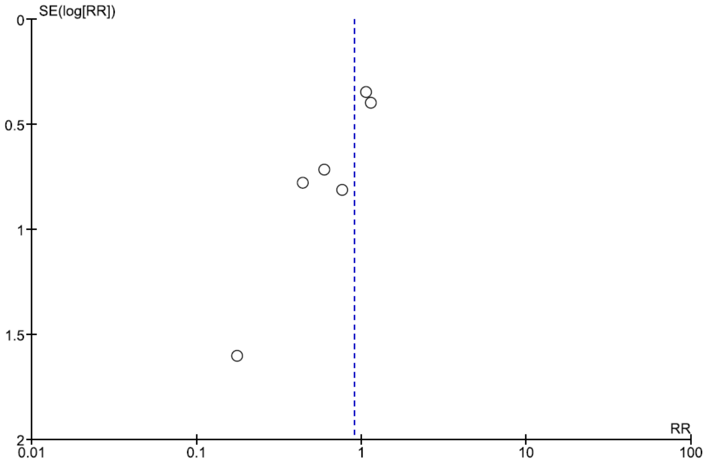

Information Classification: General

Supplemental Fig 17. Funnel of biopsy-proven acute rejection

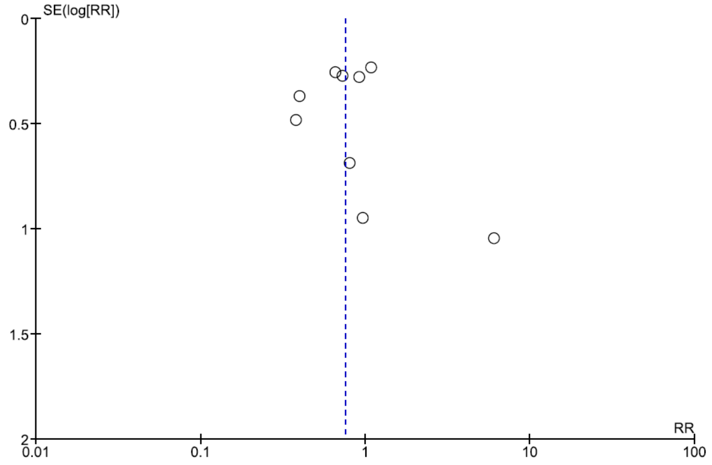

Supplemental Fig 18. Funnel of infections

a. cytomegalovirus infection

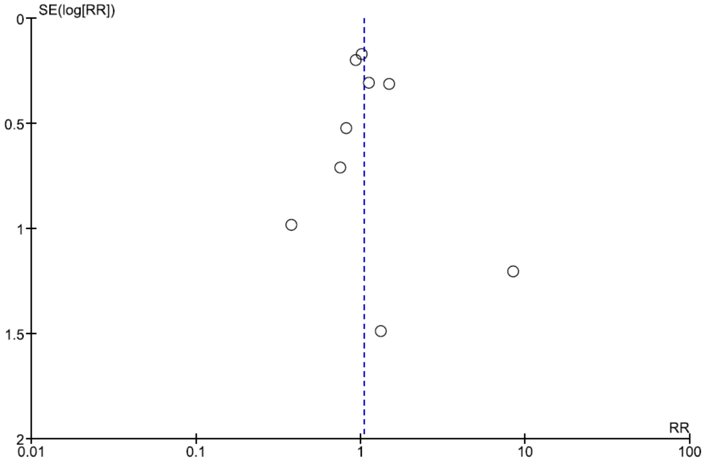

b. urinary tract infection

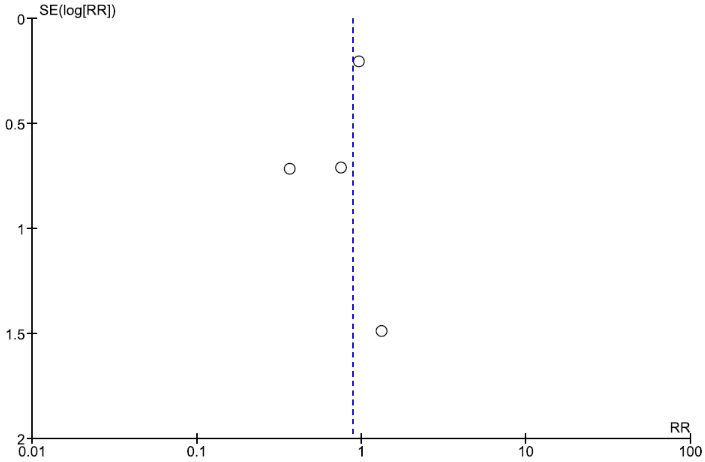

Information Classification: General

Supplemental Fig 19. Funnel of SF-36 scores

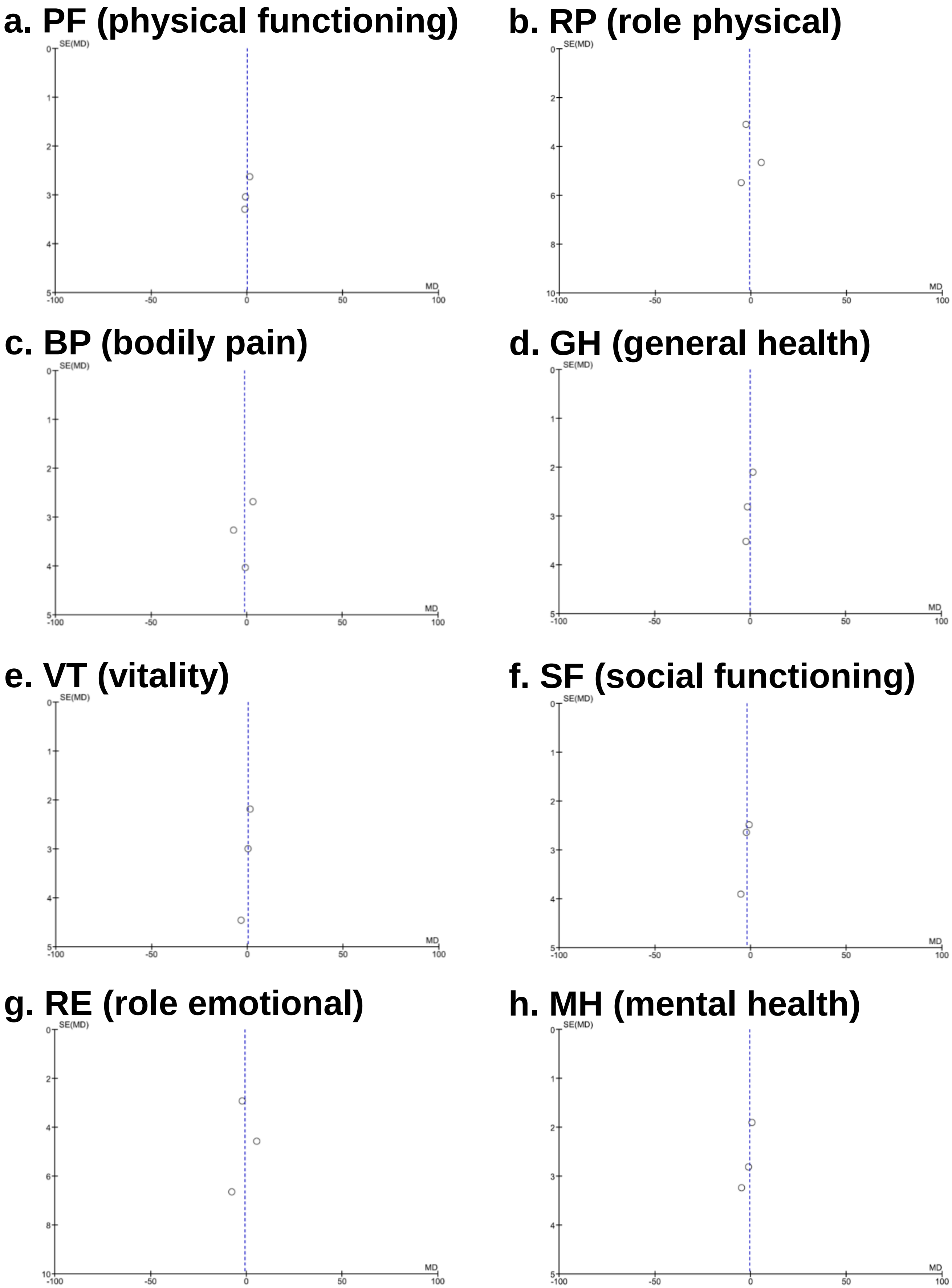

Information Classification: General
